# Architecture Design and Catalytic Activity: Non‐Noble Bimetallic CoFe/fe_3_O_4_ Core–Shell Structures for CO_2_ Hydrogenation

**DOI:** 10.1002/advs.202205087

**Published:** 2022-12-18

**Authors:** Wenkang Miao, Ronghui Hao, Jingzhou Wang, Zihan Wang, Wenxin Lin, Heguang Liu, Zhenjie Feng, Yingchun Lyu, Qianqian Li, Dongling Jia, Runhai Ouyang, Jipeng Cheng, Anmin Nie, Jinsong Wu

**Affiliations:** ^1^ Materials Genome Institute Shanghai University Shanghai 200444 China; ^2^ School of Materials Science and Engineering Zhejiang Sci‐Tech University Hangzhou 310018 China; ^3^ School of Materials Science and Engineering Xi'an University of Technology Xi'an 710048 China; ^4^ Collaborative Research Center Shanghai University of Medicine and Health Sciences Shanghai 201318 China; ^5^ School of Materials Science and Engineering Zhejiang University Hangzhou 310027 China; ^6^ Center for High Pressure Science State Key Laboratory of Metastable Materials Science and Technology Yanshan University Qinhuangdao 066004 China; ^7^ Nanostructure Research Center Wuhan University of Technology Wuhan 430070 China

**Keywords:** bimetallic CoFe, CO_2_ hydrogenation, non‐noble metal catalyst, structure–property relationship

## Abstract

Non‐noble metal catalysts now play a key role in promoting efficiently and economically catalytic reduction of CO_2_ into clean energy, which is an important strategy to ameliorate global warming and resource shortage issues. Here, a non‐noble bimetallic catalyst of CoFe/Fe_3_O_4_ nanoparticles is successfully designed with a core–shell structure that is well dispersed on the defect‐rich carbon substrate for the hydrogenation of CO_2_ under mild conditions. The catalysts exhibit a high CO_2_ conversion activity with the rate of 30% and CO selectivity of 99%, and extremely robust stability without performance decay over 90 h in the reverse water gas shift reaction process. Notably, it is found that the reversible exsolution/dissolution of cobalt in the Fe_3_O_4_ shell will lead to a dynamic and reversible deactivation/regeneration of the catalysts, accompanying by shell thickness breathing during the repeated cycles, via atomic structure study of the catalysts at different reaction stages. Combined with density functional theory calculations, the catalytic activity reversible regeneration mechanism is proposed. This work reveals the structure–property relationship for rational structure design of the advanced non‐noble metallic catalyst materials with much improved performance.

## Graphical abstract

A reversible deactivation/regeneration of the catalytic activity is found in non‐noble bimetallic CoFe/Fe_3_O_4_ core–shell structures, used for CO_2_ hydrogenation with promoted performance by rational structure design. Such phenomenon results from the oxide shell “breathing” induced by cobalt reversible exsolution/dissolution in the shell during the cycling reaction.

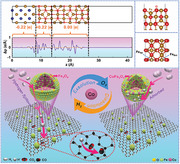

## 1 Introduction

The over consumption of fossil fuels has brought serious energy depletion and environmental issues, that is, climate change, pollution, ocean acidification, etc.,^[^ ^1^ ^]^ which severely threaten the survival of human beings, animals and plants. Developing and applying sustainable green energy in human ordinary life is urgent as an effective strategy to address the energy and environmental concerns. Catalysis plays a critical role in conversion waste gas to renewable energies, especially for the carbon dioxide (CO_2_) hydrogenation. The catalytic conversion of CO_2_, known as the reverse water gas shift (RWGS) reaction, paves the way toward the formation of value‐added chemicals (gasoline, diesel, etc.),^[^ ^2^ ^]^ and meanwhile, it can effectively ameliorate worldwide energy crisis and environmental concerns caused by massive CO_2_ emissions.^[^ ^2a^ ^]^ Unfortunately, direct reduction of CO_2_ molecule is extremely difficult due to its thermodynamical stability and chemical inertness. Besides, it generally suffers from multiply sub‐reactions and creates diverse byproducts in the hydrogenation process. Therefore, new catalysts with high activity, selectivity, stability, and low cost have been an urgent need in the industrially catalytic conversion of CO_2_.^[^ ^3^ ^]^

Metal‐based catalysts have been widely applied in various catalytic applications, resulting in both highly scientific interest and potentially industrial practices. Especially, noble metals, such as Pt,^[^ ^4^ ^]^ Au,^[^ ^5^ ^]^ Pd,^[^ ^6^ ^]^ Ru,^[^ ^7^ ^]^ etc. have been extensively studied due to their high activity. However, most noble metals have strong adsorption to CO* intermediate, which might lead to the catalysts sintering, coking and poisoning.^[^ ^8^ ^]^ Additionally, the high cost and low availability greatly limit their applications in industry catalysis. Therefore, more attentions turned toward non‐noble metal catalyst categories, including Co,^[^ ^9^ ^]^ Fe,^[^ ^10^ ^]^ and Cu.^[^ ^11^ ^]^ Their catalytic performances, that is, activity and selectivity, can be optimized to similar level as noble metals via rational structure and component design. For instance, creating surface defects,^[^ ^12^ ^]^ strong metal−support interactions (SMSI),^[^ ^11^, ^13^ ^]^ improving particle dispersion,^[^ ^14^ ^]^ etc. Among them, alloying is one of the most common and effective strategies to control the principles of economy and more importantly, to construct multicomponent systems with tunable physical and chemical properties.^[^ ^15^ ^]^ A wide range of diverse bimetallic alloy catalysts including Ni–Co alloy,^[^ ^12a^ ^]^ Ni–Au,^[^ ^15b^ ^]^ Fe–Cu,^[^ ^15c^ ^]^ Co–Fe,^[^ ^16^ ^]^ have been successfully prepared and examined for CO_2_ conversion in RWGS reaction process by far.

CoFe‐based alloy architectures were developed for CO_2_ hydrogenation with high activity and CO selectivity. Groot et al.^[^ ^16^ ^]^ found the bimetallic CoFe catalysts with Co and Fe segregating to form Janus‐like nanoparticles, displayed higher activity compared to a single element for Fisher–Tropsch (FT) reaction. Apart from the tunable properties by multi‐component system, carbon materials are often involved to effectively support and uniformly disperse catalyst nanoparticles due to their plentiful properties, including high temperatures stability (even above 750 °C under inert atmosphere), strong reducibility to metallic phases, controllable adjustment (specific surface area, defects, morphology, etc.), low price, environmental friendliness, etc.^[^ ^17^ ^]^ Meanwhile, carbon matrix can certainly inhibit the non‐synchronization of catalytic reaction process in heterogeneous catalysis by providing more active sites. Especially, defect‐rich carbon materials display excellent CO_2_ adsorption and activation ability via inducing Lewis base sites on the surface.^[^ ^18^ ^]^ Lee et al. used CoFe_2_O_4_ derivatization to obtain bimetallic alloy carbides ((Fe_1−_ *_x_* Co*_x_*)_5_C_2_) supported on the CNTs, and the heterogeneous catalysts provided higher CO_2_ hydrogenation activity than the phase‐separated Janus‐like structure of CoFe nanoparticles.^[^ ^10c^ ^]^ However, multi‐component systems might undergo out‐of‐step reactions of the metallic elements with different oxidation states, which increases the difficulty in the identification of active sites, element synergistic effects, and catalytic reaction mechanisms. Thus, atomic surface structure change and its corresponding effect on the catalytic properties of bimetallic catalyst, what is more, the bimetallic‐carbon support interaction are still unclear up to now. Deeply understanding the bimetallic catalyst structural and chemical evolution that governs the catalytic performance is the key to develop new catalysts and optimize advance catalyst with high activity.

Herein, we reported an unique nanoarchitecture of bimetallic CoFe/Fe_3_O_4_ nanoparticles with core–shell structure, loaded on defect‐rich amorphous carbon (*α*‐C) for catalytic CO_2_ hydrogenation. This nanoarchitecture exhibits enhanced activity, selectivity and stability of the RWGS reaction. Catalytic properties of different bimetallic core–shell structures, with alloy core of ≈10 nm and oxide shell of ≈2 nm, can be effectively controllable by tuning only the Co/Fe atomic ratios while the size of nanoparticles remains almost unaltered. Defect‐rich *α*‐C matrix boosts its catalytic performance by providing more active sites to absorb H_2_ and CO_2_ molecules, and simultaneously, assisting the uniform dispersion of catalyst nanoparticles. Bimetallic Co–Fe catalyst with atomic ratio of 1:1 displays the best activity, the CO_2_ conversion is 30%, and CO selectivity is over or nearly 99% at 450 °C. Moreover, it also has stable durability without conversion rate loss after being cycled by 90 h. Interestingly, we found that the reversible dissolution of Co element in iron oxide shell would result in the degradation of CO_2_ conversion, accompanying with a phase transformation from Fe_3_O_4_ to CoFe_2_O_4_. Density functional theory (DFT) calculations were as well performed to help to understand the catalytic properties of the bimetallic core–shell structures. This work provides a fundamental interpretation on the structure–activity relationship for multicomponent system of advanced non‐noble metal catalysts in great potential energy and environmental applications.

## 2 Results and Discussion

### 2.1 Synthesis and Characterization of the Bimetallic CoFe/Fe_3_O_4_ Core–Shell Structures

**Figure 1:**
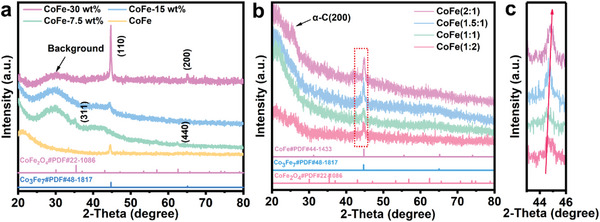
Phase characterization of bimetallic alloy catalyst. a) The XRD patterns of CoFe with different loading amounts. b) The XRD patterns of CoFe with different Co/Fe atomic ratios (loading mass is 15 wt%). c) Enlarged view of the dashed frame in (b).

The bimetallic CoFe/Fe_3_O_4_ core‐shell structures formed on the defect‐rich *α*‐C substrate by a specific thermal reduction reaction of CoFe_2_O_4_ nanoparticles loaded on layered g‐C_3_N_4_ nanosheets in the mixed reaction gas (25 vol% H_2_, 25 vol% CO_2_, and 50 vol% Ar) at 450 °C (Figure S1, Supporting Information). The composite catalyst structures have been fully investigated by X‐ray diffraction (XRD) patterns in **Figure** **1**. Two diffraction phases are noteworthily identified in low loading amount percentage of 7.5 wt% on *α*‐C, the diffraction peaks at 44.4° and 65.0° correspond to the (110) and (200) of Co_3_Fe_7_ alloy, and the diffraction peaks at 35.2° and 62.4° belong to the (311) and (440) of CoFe_2_O_4_, respectively. It implies that CoFe_2_O_4_ is partially reduced in H_2_ and then oxidized in air. While increasing the loading mass to 15 wt%, only a single phase of Co_3_Fe_7_ can be detected by XRD patterns. By tuning the atomic ratios of Co and Fe in precursors, a phase transformation from Co_3_Fe_7_ to CoFe occurs with same loading amount, as shown in Figure 1b. The diffraction peak of the (100) gradually shifts to larger angles as labelled by red arrow in Figure 1c, with increasing the content of Co. Thermal treated temperatures also markedly influence the components of composite catalyst. As temperature increases to 500 °C, all oxide nanoparticles transit to pure bimetallic alloy phase (Figure S2, Supporting Information). Notably, the crystal g‐C_3_N_4_ nanosheets (Figure S1a–c, Supporting Information) have been totally transformed to amorphous carbon after the specific thermal treatment, with nearly no diffraction peaks of carbon being observed in the XRD patterns (Figure 1; Figure S2, Supporting Information).

**Figure 2:**
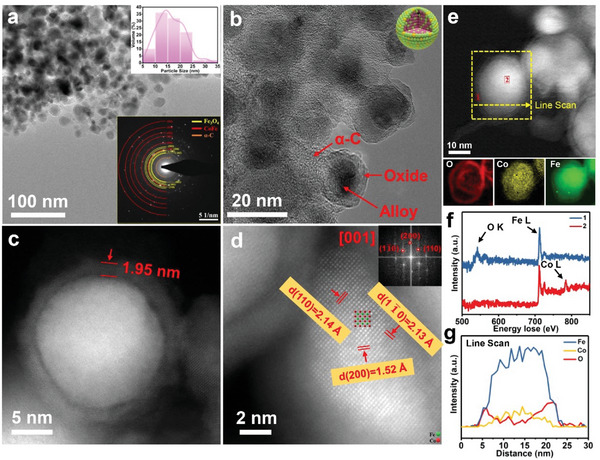
Characterization of CoFe (1:1) (CoFe/Fe_3_O_4_). a,b) Low magnification TEM images of the catalysts, the insets respectively show the SAED and particle size distribution. c,d) AC‐STEM images of an individual nanoparticle, the inset on the upper right is FFT image of the selected area. e) EELS mapping. f) Core‐loss spectrums of O K‐edge, Fe L‐edge, and Co L‐edge. g) EELS line scan spectrum along the dash line in (e).

The fundamental morphologies and structures of bimetallic CoFe catalysts with different loading amount, have been investigated by aberration corrected scanning transmission electron microscopy (AC‐STEM) in **Figure** **2**. The average particle size of bimetallic CoFe (1:1) nanoparticles on *α*‐C (loading amount of 15 wt%) is ≈15 nm (the inset in Figure 2a). Two phases are clearly identified by selected area electron diffraction (SAED) patterns, that is, CoFe alloy and Fe_3_O_4_ (Figure 2a; Figure S3, Supporting Information), which agree well with the XRD results above. A typical core–shell structure has been founded in Figure 2b, with core size of decades of nanometers and shell thickness of ≈1.95 nm (Figure 2c). The core–shell nanoparticles evenly embed in the *α*‐C substrate without agglomeration. Interestingly, the loading amounts on *α*‐C substrate have an important effect on the bimetallic alloy size distribution. As shown Figures S4–S6, Supporting Information, the particle size of CoFe becomes larger compared with *α*‐CoFe‐15 wt%, regardless the loading amounts increase or decrease. Additionally, carbon support shows strong size regulation. The average particle size would increases significantly to ≈35 nm as the absence of carbon support (Figure S4d, Supporting Information), which is over two times larger than that on *α*‐C under the same loading amount. That implies the confinement growth of nanoalloy catalysts on carbon supports that play a vital role in effectively preventing high temperature sintering and improving stability of the catalyst materials. Simultaneously, the electron beam irradiation will not damage the sample morphology and phase in Figure S7, Supporting Information. The atomic resolution high‐angle annular dark field (HAADF) image in Figure 2d, reveals the atomic structure of the core region, which can be well indexed as the [001] zone axis of CoFe alloy by the corresponding fast Fourier transform (FFT) as inset of Figure 2d. However, a certain degree of lattice expansion in the core region is found compared to its theoretical lattice parameters (Table S1, Supporting Information), it is possibly induced by the lattice mismatch between the shell and core.

To further understand the chemical composition of the core–shell structure, we carried out electron energy loss spectrum (EELS) mapping on the dotted square in Figure 2e–g. It can be clearly shown that the main components of shell region (position 1) are iron oxides. In the core (position 2), nearly no oxygen element is observed, while elements of Co and Fe are rich as evident from the spectra of O K‐edge, Fe L‐edge, and Co L‐edge (Figure 2f). Corresponding EELS mapping for O, Fe, and Co are shown in the bottom of Figure 2e and the line scan profiles along the yellow dashed arrow (Figure 2g) further confirm the compositions of core–shell structure of each nanoparticle, that is, the shell is composed by iron oxides, and the core is proved as CoFe alloy.

### 2.2 Carbon Dioxide Hydrogenation Performance

**Figure 3:**
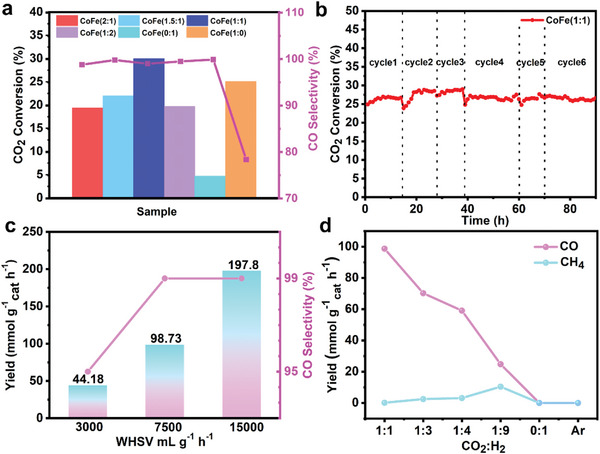
Comparison of catalytic activities for atmospheric pressure CO_2_ hydrogenation. a) CO_2_ hydrogenation activity of *α*‐C supported CoFe/Fe_3_O_4_ with different Co/Fe atomic ratio at 450 °C. b) CO_2_ hydrogenation stability of CoFe (1:1) at 450 °C. c) CO_2_ hydrogenation activity at different WHSV, the stick represents CO_2_ conversion and the dot‐line graph represents CO selectivity. d) CO_2_ hydrogenation activity at different CO_2_/H_2_ ratio.

The catalytic performance of bimetallic CoFe/Fe_3_O_4_ for RWGS reaction at atmospheric pressure has been comprehensively investigated to evaluate the catalytic activity for CO_2_ reduction. **Figure** **3**a shows CO_2_ conversion and CO selectivity of the bimetallic catalysts with different atomic ratios of Co and Fe. Surprisingly, as the atomic ratio is 1:1, it shows appealing catalytic activity with CO_2_ conversion of 30% and CO selectivity of ≈99% compared to those with other ratios, and even among the widely explored catalysts (Table S2, Supporting Information). Unlike the single‐metal catalysts of pure Co or Fe, the bimetallic catalysts exhibit remarkable tunability in terms of activity. The CO_2_ conversion rate and CO selectivity of are 4.7% and 99% of pure Fe, while 25.4% and 78.4% of pure Co (Figure 3a), respectively, although the structures and morphologies of both them keep similar with those of bimetallic alloys (Figures S8,S9, Supporting Information). Therefore, optimized catalyst performance is envisaged with a tailored component, structure, and working conditions (Figure 3a; Figure S10, Supporting Information). Fe_3_O_4_ surface is in favor of providing high activity in CO_2_ hydrogenation reaction,^[^ ^19^ ^]^ the unique bimetallic alloy/iron oxide core–shell structure contributes to the activation of reactive gases and the optimum adsorption energies of intermediates through the redistribution of catalyst electrons.

Figure 3b shows the *α*‐C supported bimetallic CoFe/Fe_3_O_4_ core–shell catalysts have long‐term durability with CO_2_ conversion rate retention of 27% after 90 h/6 cycles. It indicates the structures of bimetallic nanoparticles maintain intact without any dramatical agglomeration or sintering as exposure to high temperature for a long time, under the protection of the Fe_3_O_4_ thin shell. Interestingly, we found that activity deterioration of such catalyst during the first 3 h in each cycle, and the CO_2_ convention rate almost drops to 80% of that stable value. After that, the performance will be restored to its original level. The catalytic rate remains basically unchanged and the selectivity is improved as the WHSV increases (Figure 3c), again proving excellent stability of such catalysts. When the volume ratio of CO_2_ and H_2_ decreases, the catalytic activity decreases, while CH_4_ selectivity increases (Figure 3d), because excess H_2_ leads to further hydrogenation. When switched to Ar as reactant gas, no CO or CH_4_ can be detected, indicating the syngas production originates from CO_2_ reduction rather than being decomposed by the catalyst (Figure 3d).

### 2.3 Activity Deterioration and Restoration Mechanism

**Figure 4:**
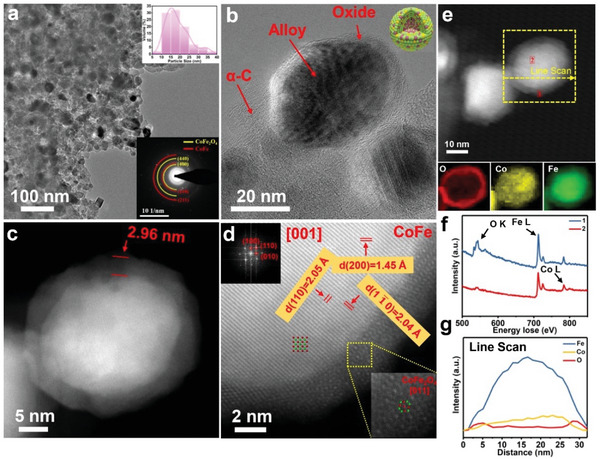
Deactivation of catalyst and dissolution of Co into shell (CoFe/CoFe_2_O_4_). a,b) TEM images of CoFe (1:1) reacted for 40 h after the third cycle, the inset shows the SAED and particle size distribution. c,d) AC‐STEM images of an individual nanoparticle. e) EELS mapping. f) Core‐loss spectrums of O K‐edge, Fe L‐edge, and Co L‐edge. g) EELS line scan spectrum along the dash line in (e).

To deeply explore the deactivation and restoration mechanism, atomic structures of the catalysts cycled for 40 h and fully oxidized in air are characterized by AC‐STEM. Upon reaction in the first 3 hours, the activity of CoFe/Fe_3_O_4_ catalysts have markedly dropped down. Analogously, the catalyst remains similar nanoparticle morphology with same average size of ≈15 nm (**Figure** **4**a), as those before reaction. However, a phase transformation occurs in the shell from initial Fe_3_O_4_ to CoFe_2_O_4_, as identified by SAED pattern (inset in Figure 4a) collected from the catalysts after three cycles for 40 h and fully oxidized in air. Although the nanoparticles are uniformly embedded in the amorphous carbon matrix, their shell thickness becomes noticeably larger (2.96 nm) than its initial state (1.95 nm), as presented in Figure 4b,c. The STEM image contrast of shell is much darker than that of core, it implies the atomic number of the shell components is lighter. The shell phase is theoretically indexed as CoFe_2_O_4_, and the core phase still is CoFe, according to the atomic‐scale lattice analysis in Figure 4d. Moreover, due to the exsolution of Co element from the internal, the lattice expansion of the CoFe alloy is significantly reduced (Table S3, Supporting Information).

The elemental composition and structure of the sample are also disclosed by EELS (Figure 4e–g). Comparing to the sample before cycling (Figure 2e–g), migration of Co into the shell is observed in the cycled sample and fully oxidized in air. It can be seen that Fe element is observed in both shell and core of the nanoparticle (outlined by the dashed square in Figure 4e), and oxygen mainly distributes in the shell part, which is the same as the pristine catalyst. However, Co L‐edge signals are detected in both shell and core region, where in the pristine sample Co is found only in the core. It indicates Co partially migrates from core to shell with forming the spinel CoFe_2_O_4_ as exposure in the air. This Co dissolution has a detrimental effect on the catalyst activity. After activation exposed to the reaction atmosphere again, Co will in turn, migrates back to the CoFe alloy, where the catalytic activity is regenerated. The reversible exsolution/dissolution of Co in the shell was confirmed again by the shell breathing behavior with thickness periodical alternation in cycles (Figures S11,S12, Supporting Information).

**Figure 5:**
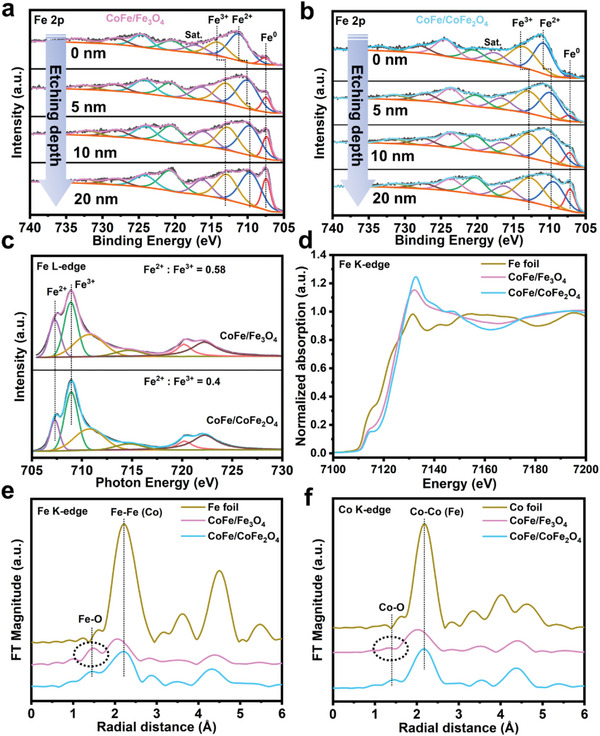
Electronic structural characterization of activity degradation and regeneration. The high‐resolution Fe 2p XPS depth profiling of a) CoFe/Fe_3_O_4_ and b) CoFe/CoFe_2_O_4_. c) Fe L‐edge XAFS of CoFe/Fe_3_O_4_. d) Normalized Fe K‐edge XANES of CoFe/Fe_3_O_4_. e,f) Corresponding Fourier transforms of the *k* ^3^‐weighted EXAFS at the Fe K‐edge and Co K‐edge.

The electronic structure and valence state of the catalysts were characterized by X‐ray photoelectron spectroscopy (XPS) and X‐ray absorption fine structure (XAFS), when Co is migrated into versus out of the shell, as shown in **Figure** **5**, Figure S13 and Table S4, Supporting Information. The content of zero‐valent metals gradually increases with deepening the etch depth in all samples (Figure 5a,b; Figure S13a,b, Supporting Information), which implies a core–shell structure feature of an oxide surface (shell) and an alloy interior (core). The internal alloy is susceptible to be oxidized by comparing Figure 5a,b and Figure S13a,b, Supporting Information, when the sample is exposed to air. Table S4, Supporting Information, summarizes the proportion of Fe and Co in different valence states before and after Co migration, quantified by peak fitting of XPS depth profiles. It is obvious that the proportion of Fe^2+^ in CoFe/Fe_3_O_4_ is significantly higher than that of CoFe/CoFe_2_O_4_, especially at the depth of 20 nm. What is more, the content of Co^0^ is always more than Fe^0^ at almost any depth and reaction state. Combined with XRD phase analysis before and after the single element reaction (Figure S8, Supporting Information), it can be concluded that Fe_3_O_4_ is a relatively stable, but Co is susceptible to be oxidized and reduced under different reaction states. The Fe L_2_‐edge and L_3_‐edge of XAFS emerge obvious splitting in Figure 4c, implying that Fe is in an oxidized state,^[^ ^20^ ^]^ the shoulder peak on the left indicates the presence of Fe^2+^. Therefore, we can draw a conclusion that the content of Fe^2+^ is relatively high in the sample before oxidation (CoFe/Fe_3_O_4_), which is mainly contributed by Fe_3_O_4_. The lower chemical state of the sample before oxidation is further confirmed in Fe k‐edge XANES (Figure 5d). The corresponding extended X‐ray absorption fine structure (EXAFS) spectra revealed the formation of new Co—Fe bonds^[^ ^21^ ^]^ in Figure 5e,f. Significantly, obvious Fe—O bond appears in the spectrum, while Co—O bond almost can be negligible in the sample of CoFe/Fe_3_O_4_,^[^ ^22^ ^]^ which again proved that the shell is composed by Fe_3_O_4_ rather than CoFe_2_O_4_ in the reaction state, and Co mainly exists more in the form of metal. This XAFS results are consistent with those of XPS depth profiling. Therefore, we propose a structure–activity relationship that the reversible exsolution/dissolution of Co into the shell induced by the reaction environment lead to the activity deterioration and restoration.

### 2.4 Regulation of Core–Shell Structural Components

**Figure 6:**
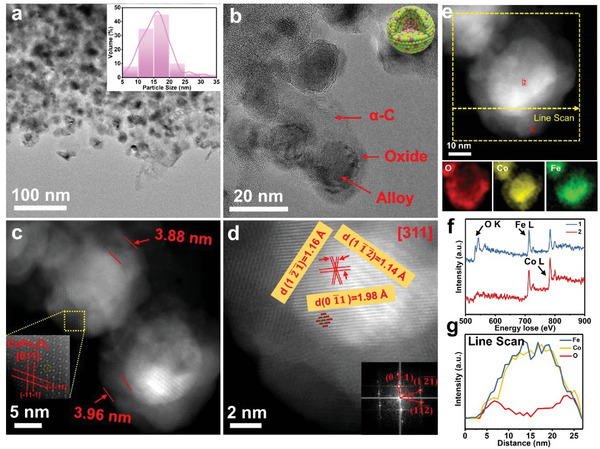
Regulation of core–shell structural components. a,b) TEM images of CoFe (2:1), the inset shows the SAED pattern and particle size distribution. c,d) AC‐STEM images of CoFe (2:1). e) EELS mapping. f) Core loss spectra of O K‐edge, Fe L‐edge, and Co L‐edge. g) EELS line scan spectrum traced along the yellow dotted lines.

Structural component modification largely enhances the capability of tuning the catalytic performance. Therefore, in order to have more knowledge of influence of Co appearance in oxide shell on catalytic activity, the atomic ratio of Co continually is increased to 2:1 versus Fe in bimetallic catalysts, and the microstructure after cycling are detailly investigated by using AC‐STEM combined with EELS as shown in **Figure** **6**. There is no significant change in the particle size distribution and morphology in comparison to that of Co:Fe (1:1). Such catalyst nanoparticles are dispersed on the amorphous carbon substrate with average size of 15 nm (Figure 6a,b). However, it is interesting that the thickness of the oxide shell increases from 1.9 to 3.9 nm (Figure 6c), indicating more Co appears in the outer shell, the expansion in CoFe alloy is released by directly measuring *d*‐spacing (Figure 6d; Table S5, Supporting Information). Fortunately, the phases of the oxide layer are identified as spinel‐like CoFe_2_O_4_, based on indexing the lattice structure and FFT. Similarly, Co element is clearly observed in both shell and core region through EELS maps (Figure 6e) and the corresponding core‐loss spectra for O K‐edge, Fe L‐edge, and Co L‐edge in position 1 and 2 (Figure 6f), respectively. Accordingly, we conclude that the dissolved of Co and the thickened spinel‐like oxide shell of bimetallic catalyst should be the main reason for the partial loss of catalytic activity.

We have evaluated the stability of the oxide outer in the reaction atmosphere, the XRD patterns show that hydrogen‐treated (25 vol% H_2_, 75 vol% Ar) samples are still *α*‐C and CoFe alloy, and the diffraction peaks of 27.0° and 44.4°, can be attributed to (200) of *α*‐C and (110) of CoFe, respectively (Figure S14, Supporting Information). After reduction, the nanoparticle size looks smaller (10 nm) than the sample pretreated in reaction gas, while its microstructure keeps unaltered that core–shell structural bimetallic catalyst particles are still evenly embedded in carbon support, as shown in Figure S15, Supporting Information. In addition, the thickness of the shell visibly decreases to 1.26 nm in where no identification of Co element from EELS maps in Figure S16, Supporting Information.

### 2.5 Regulation of the Matrix

**Figure 7:**
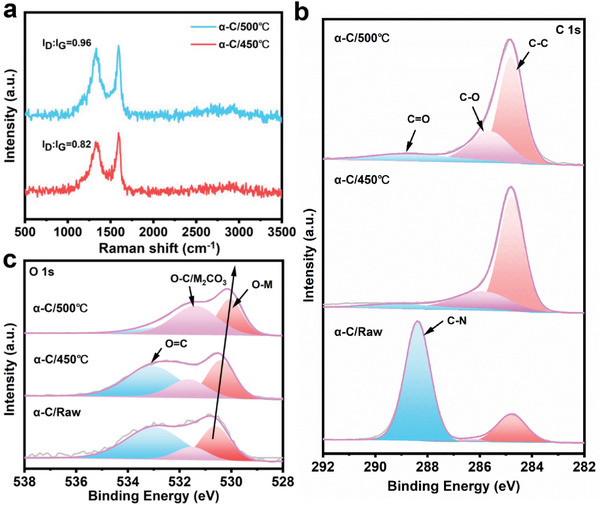
Regulation of the matrix. a) The Raman spectra of *α*‐C pretreated at 450 °C and 500 °C. The corresponding high‐resolution XPS spectra of b) C 1s and c) O 1s.

The superiority of *α*‐C and interactions between bimetallic catalyst and matrix have been deeply explored by comparison to different kinds of supports, for example, TiO_2_ and MXene. The XRD and SEM results indicate that bimetallic Co_3_Fe_7_ (size in 15–30 nm) was successfully modified on TiO_2_ and MXene supports (Figures S17,S18, Supporting Information). Relative performance tests present that *α*‐C matrix shows the best comprehensive performance (CO_2_ conversion and CO selectivity) among different supports, thereinto, the activity of *α*‐C supported bimetallic catalyst is 2.2 times higher than that on MXene and 1.4 times higher than that on TiO_2_ (Figure S19, Supporting Information). Raman spectrum presents that there are abundant defects in *α*‐C, and defect density increases with rising pretreatment temperature (from 0.82 to 0.96 of *I*_D_:*I*_G_) (**Figure** **7**a). It is noteworthy that pure g‐C_3_N_4_ is maintained stably in both structure and morphology during the reaction conditions (Figures S20,S21, Supporting Information), meaning that Co/Fe element can catalyze the conversion of g‐C_3_N_4_ to defect‐rich *α*‐C. XPS is used to obtain the electronic state of the bimetallic catalyst, as shown in Figure 7 and Figure S22, Supporting Information. The peak at 285.7 eV is attributed to the C—O bond in the C 1s region, it should be attributed to the adsorption of CO_2_ on the *α*‐C defect sites, providing reasonable evidence for significant enhancement of the absorption of CO_2_ in the abundance defective carbon. The C—O bond (531.5 eV) caused by oxygen adsorption has been found in the O 1s region. Simultaneously, CO_2_‐temperature programmend desorption (CO_2_‐TPD) was performed to illustrate the effect of reactive gas adsorption of different supports on catalytic activity, as shown in Figure S23, Supporting Information. There were three categories for the basic sites including weak (temperature below 100 °C), moderate (temperature between 100 °C and 150 °C), and strong (temperature above 150 °C), *α*‐C/CoFe has much higher CO_2_ adsorption in the moderate region than the other supports, which is attributed to the abundant Lewis base sites provided by the defects.^[^ ^18^ ^]^ This once again confirmed that the defect‐rich *α*‐carbon substrate contributes to the adsorption of CO_2_. Moreover, the amount of bimetallic CoFe alloy increases gradually with the rise of reduction temperature (Figure S24, Supporting Information), which is consistent with the results of XRD (Figure S2, Supporting Information). XPS and CO_2_‐TPD combined with Raman results demonstrate that the defect‐rich amorphous carbon support is favor to improve the adsorption and activation capacity of CO_2_ and further enhance the catalytic activity.

### 2.6 Catalytic Reaction Mechanism

**Figure 8:**
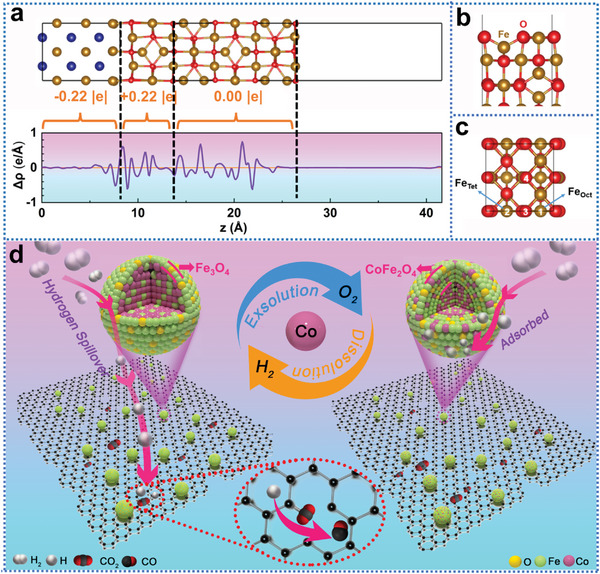
Catalytic reaction mechanism. a) Slab model of CoFe/Fe_3_O_4_ core/shell structure, and the charge density difference: Δ*ρ*_CoFe/oxide_ = *ρ*_CoFe/oxide_ − *ρ*_CoFe_ − *ρ*_oxide_. The amount of transfer electron at the interface are indicated. b) Side view of the Fe_3_O_4_ (100) surface. c) Top view of the Fe_3_O_4_ (100) surface unit cell. The numbers at the top of the atoms indicate the different adsorption sites. d) Schematic diagram of the reaction mechanism.

To check the influence of core–shell structure on its catalytic activities, DFT calculations were performed on the highly active CoFe/Fe_3_O_4_ core–shell structure and the comparatively inert Fe_3_O_4_ oxide. Comparing between unsupported CoFe_2_O_4_ and Fe_3_O_4_, H atom has stronger adsorption (−3.95 eV) on the former surface than on the latter (−3.40 eV), the corresponding adsorption energy values are shown in Table S6, Supporting Information. This is because that the Co atom has higher electronegativity than Fe, which leads to less electron transfer from Co than Fe to O atoms, and thus the O atoms in CoFe_2_O_4_ tends to bind H atoms stronger than that in Fe_3_O_4_. The H adsorption on CoFe_2_O_4_ is so strong that is will further cause difficulty of H desorption, and thus less active on CoFe_2_O_4_ for the considered reaction. That should mainly account for high catalytic activity of the bimetallic CoFe/Fe_3_O_4_ core–shell catalysts. In addition, the formation energy of oxygen vacancy on CoFe_2_O_4_ (4.67 eV) is about 0.5 eV less than on Fe_3_O_4_ (5.15 eV) because of weaker bond of Co—O than Fe—O. However, it is thermodynamically unfavorable (bonding energy of O_2_ is 3.0 eV per O atom) for the formation of oxygen vacancies to release O_2_ on both CoFe_2_O_4_ and Fe_3_O_4_,

**Figure** **8**a shows charge density difference that charge transfer occurs from the metal core to the oxide at the interface with forming metal—oxygen bonds. Such charge transfer is mainly localized near the interface by the oxygen atoms gaining electron from the metals, whereas no net charge transfer was found after the thickness of about five atomic layers. Therefore, we expect the influence of the interfacial charge transfer on the surface chemical properties of for pure bimetallic alloy core–shell catalysts can be neglected when the oxide thickness is greater than 1 nm. Considering the internal lattice strain induced by expanded lattice (8.57 Å) in Fe_3_O_4_ outer layer, compared to perfect Fe_3_O_4_ crystal (8.39 Å),^[^ ^23^ ^]^ we had compared the surface properties of Fe_3_O_4_ in Table S6, Supporting Information. Taking H as the probe atom, it was found the adsorption energy at the most favorable sites atop O is slightly stronger on the expanded oxide surface. Also, results show the surface oxygen formation on the expanded oxide looks slightly easier than on the normal oxide surface. Overall, the change of surface properties due to the lattice expansion (0.2 Å) is not the significant reason for the bimetallic CoFe/Fe_3_O_4_ core–shell catalysts.

In order to check again the CO_2_ adsorption on the catalyst surface, the sample was first treated in a pure CO_2_ environment, then driven out all CO_2_ in reactor by Ar, and then H_2_ began to be fed until the left CO_2_ had been completely reacted. Surprisingly, we detect the existence signal of CH_4_ but not CO (Figure S25a, Supporting Information), indicating there are plenty of CO_2_ have adsorbed on the surface of catalyst powders and hydrogenated to CH_4_ when hydrogen is fed into the reaction process. As reported, CO_2_ can be physically adsorbed on Fe_3_O_4_ (001), and it is easily desorbed.^[^ ^24^ ^]^ Thus, it should be that defect‐rich amorphous carbon substrate provides more active sites for excess CO_2_ adsorption and activation to the Lewis base sites, assisting to boost the catalytic performance. And the selectivity tends to CH_4_ due to the lack of hydrogen assisted in the CO_2_ activation process, resulting in the production of intermediates conducive to methanation. Controversially, if the sample was first treated in H_2_, following by removing all H_2_ in reactor via Ar, and then CO_2_ began to be fed up till H_2_ was completely depleted. A large amount of CO is identified, yet the yield of CH_4_ is 0 (Figure S25b, Supporting Information), implying H_2_ is dissociated and spilled out on the metal, which then assists in the activation and conversion of CO_2_ on the defective carbon support.

Consequently, we rationally speculate the reaction mechanism based on experimental and theoretical analysis above. As shown in the schematic diagram of Figure 8d, H_2_ will be dissociated on the CoFe/Fe_3_O_4_ surface, and meanwhile, hydrogen will spillover onto the defect‐rich amorphous carbon substrate that assists the activation and conversion of CO_2_ via adsorption the reactive gas. The interplay of the bimetallic catalyst with *α*‐C has exceptional importance in the catalyst activity. However, too strong hydrogen adsorption on CoFe_2_O_4_ hinders the spillover of hydrogen onto the carbon support, finally, inducing a slightly drop in activity, and the activity can be recovered as the adsorbed hydrogen reduces CoFe_2_O_4_ to Fe_3_O_4_.

## 3 Conclusions

In summary, we successfully synthesized defect‐rich *α*‐C supported non‐noble bimetallic CoFe/Fe_3_O_4_ core–shell nanoparticles via a simple and versatile route, and deeply reveal the catalytic reaction mechanism through fine structure characterization by using AC‐STEM. The catalysts exhibit advantageous performance, that is, CO_2_ convention rate of 30% and CO selectivity nearly or over of 99% at 450 °C, during CO_2_ hydrogenation process, through tunable ensemble composition and loading amount versus carbon support. The bimetallic alloy catalysts display excellent stability with activity retention remaining almost of 100% after cycling for 90 h. Notably, the exsolution/dissolution of Co in the oxide layer leads to the surface change of bimetallic alloy nanoparticles, including atomic structure and electronic property, which accounts for the activity deterioration and restoration in each cycle. The spinel‐like CoFe_2_O_4_ formation in the shell region makes partial activity loss, due to relatively stronger hydrogen adsorption. Additionally, defect‐rich *α*‐C support is favored to providing active sites for the reactive gas adsorption that greatly boosts the catalytic activity and selectivity in the overall reaction with the spillover hydrogen assisted activation. This work deepens the understanding on relationship of structure features and catalytic properties, and provides fundamental insights into bimetallic catalyst for rational design of microstructures with tunable and well‐controlled properties.

## 4 Experimental Section

### Materials

Cobalt (II) acetylacetonate (98%), iron (III) acetylacetonate (97%), triethylene glycol (99%), and ethyl acetate (99%) were purchased from J&K Scientific. Urea (98%) was purchased from Sinopharm Chemical Reagent Co., Ltd. All chemicals were used here without further purification.

### Synthesis of Bimetallic CoFe/Fe_3_O_4_ Core–Shell Structures

Porous g‐C_3_N_4_ nanosheets were first prepared according to a typical two‐step thermal polymerization method by using urea as precursor.^[^ ^25^ ^]^ Secondly, Co*_x_* Fe_2−_ *_x_* O_4_ nanoparticles were synthesized through a simple wet‐chemistry procedures,^[^ ^26^ ^]^ and the tunability of composition can be achieved by change the molar ratios of cobalt and iron salt precursors, that is, 2:1, 1.5:1, and 1:1, details are in the Supporting Information. Then prepared g‐C_3_N_4_ nanosheets and Co*_x_* Fe_2−_ *_x_* O_4_ nanoparticles were respectively weighed and dispersed in water by ultrasonic to obtain a uniform dispersion. During ultrasonication, 15.3 mL of 1.5 mg mL^−1^ CoFe_2_O_4_ nanoparticle dispersion was added dropwise to 100 mL of 1.3 mg mL^−1^ g‐C_3_N_4_ nanosheet dispersion, continued to be ultrasonicated for 1 h, and freeze‐dried to obtain a CoFe_2_O_4_ content of 15 wt%. The g‐C_3_N_4_/CoFe_2_O_4_ nanocomposite are obtained. Different loading amount specimen could be obtained by changing the added volume of CoFe_2_O_4_ dispersion. Finally, a series of bimetallic CoFe samples were obtained by pretreatment in a reaction atmosphere. The sample obtained after pretreatment was marked as CoFe (X:Y), where X/Y represents the atomic ratio of Co/Fe. The different substrate synthesis strategy, for example, TiO_2_ and MXene, shared the same merit of experimental parameters with g‐C_3_N_4_.

### Catalyst Characterization

The phase structure was identified by powder X‐ray diffraction (XRD, Bruker‐D2, Germany) with Cu K*α* radiation (*λ* = 1.5418 Å). The morphology images were collected by a field emission scanning electron microscope (SEM, Hitachi SU8230, Japan). The electronic structure was obtained by X‐ray photoelectron spectrometer (Escalab 250Xi, Thermo Scientific, America). The high‐resolution transmission electron microscopy (HRTEM) images and atomic HAADF STEM images were characterized by utilizing Cs‐corrected transmission electron microscope (Titan G3 80–300 kV, Thermo Fisher Scientific, USA), which was operated at 300 kV. The EDS and EELS measurements were characterized by related detectors configured in the STEM system. Soft and hard XAFS were recorded at Shanghai Synchrotron Radiation Laboratory (BL02B02). CO_2_‐TPD is performed via an integrated resonant micro‐cantilever and developed into micro‐electromechanical system‐based TGA (MEMS TGA, High‐End MEMS Technology Co., Ltd., China).

### Catalyst Evaluation for the RWGS

The catalytic performance of catalyst RWGS was evaluated in a continuous fixed‐bed quartz reactor (*d* = 6 mm) under atmospheric pressure. Typically, 20 mg of catalyst was placed between two layers of quartz wool in the center of the reactor tube. Before the catalytic test, the catalyst was pretreated at 450 °C for 3 h with reaction gas (H_2_/CO_2_/Ar = 2.5/2.5/5 mL min^−1^, WHSV = 30 000 mL h^−1^ g cat^−1^) under atmospheric pressure. Subsequently, the performance at different temperatures (300–500 °C) was tested under the same feed gas conditions. The product was analyzed by an online gas chromatograph (GC7900II, Tianmei, China) equipped with TDX‐01 column, a thermal conductivity detector, and flame ionization detector. The CO_2_ conversion and CO selectivity were calculated by Equations (1) and (2).

(1) $$CO_{2}\;\mathrm{conversion}\;\left(\%\right)=\frac{CO_{2,\mathrm{in}}-CO_{2,\mathrm{out}}}{CO_{2,\mathrm{in}}}\times 100$$

(2) $$\mathrm{CO}\;\mathrm{selectivity}\;\left(\%\right)=\frac{{\mathrm{CO}}_{\mathrm{out}}}{{\mathrm{CO}}_{\mathrm{out}}+{\mathrm{CH}}_{4,\mathrm{out}}}\times 100$$

### DFT Methods

Spin‐polarized DFT calculations were performed by using the Vienna Ab initio Simulation Package (VASP),^[^ ^27^ ^]^ with the Perdew−Burke−Ernzerhof (PBE) functional under the generalized gradient approximation,^[^ ^28^ ^]^ and the projected augmented wave (PAW) pseudopotentials.^[^ ^29^ ^]^ The GGA+U approach by Dudarev et al.^[^ ^30^ ^]^ was employed to treat the on site Coulomb interactions on the localized 3d electrons of Fe and Co, with the U values of 4.0 and 3.3 eV, respectively, taken from the ref. [31]. The energy cutoff of 450 eV was used for the plane‐wave basis set, and the 5 × 5 × 1 mesh was used for the *k*‐points sampling in all the calculations.

The core–shell structure of CoFe/Fe_3_O_4_ was modeled by a 1 × 1 unit cell of Fe_3_O_4_ (100) of 17‐atom‐layer thickness supported on top of the 2 × 2 unit cell of CoFe (100) alloy surface with 6‐atom‐layer thickness. According to the measured distance of (110) on the CoFe/Fe_3_O_4_ surface of 2.14 Å, the lattice vector length of the slab model of CoFe (100)/Fe_3_O_4_ (100) was 6.06 Å. Here, the “6.06 Å” was corresponding to the lattice constant of 8.57 Å for Fe_3_O_4_ shell of cubic phase, which was slightly expanded compared to experimental value of 8.394 Å in perfect Fe_3_O_4_ crystal. As a result, the thickness of the 17‐atom‐layers Fe_3_O_4_ (100) had about 1.9 nm, which was comparable to that of the 1.95 nm in the CoFe/Fe_3_O_4_ system found in the experiments. In modeling the CoFe_2_O_4_ (100) surface, all the Fe atoms at the tetrahedron sites were replaced with Co atoms. The magnetic ordering in the CoFe alloy was set to ferromagnetic, and in Fe_3_O_4_ and CoFe_2_O_4_ the metal atoms at the octahedral sites were antiferromagnetically coupled to those at the tetrahedral sites, details are in the Supporting Information.

In the structure optimization of Fe_3_O_4_ (100) and CoFe_2_O_4_ (100), all atoms were allowed to relax until the residual forces on each atom are less than 0.03 eV Å^−1^. For CoFe/Fe_3_O_4_, the bottom four layers of the CoFe metal atoms were fixed to their bulk positions, and all other atoms were allowed to relax. Afterward, in the calculation of H atom adsorption and O vacancy formation, only the top four layers of the oxide surfaces were allowed for further relaxation. The surface adsorption energy of H atom, $\Delta E_{\mathrm{ads}}^{H}$, and oxygen vacancy formation energy, $\Delta E_{\mathrm{vac}}^{O}$, were respectively defined as:

(3) $$\Delta E_{\mathrm{ads}}^{H}=E_{\mathrm{slab}+H}-E_{\mathrm{slab}}-E_{H}$$

(4) $$\Delta E_{\mathrm{vac}}^{O}=E_{\mathrm{slab}+V_{O}}+E_{O}-E_{\mathrm{slab}}$$

## Conflict of Interest

The authors declare no conflict of interest.

## Supporting information

Supporting Information

## Data Availability

The data that support the findings of this study are available from the corresponding author upon reasonable request.
