# Supplementary material for: Architecture Design and Catalytic Activity: Non‐Noble Bimetallic CoFe/fe3O4 Core–Shell Structures for CO2 Hydrogenation
Source: Adv Sci (Weinh). 2022 Dec 18;10(5):2205087. doi: 10.1002/advs.202205087 (PMC9929264; doi:10.1002/advs.202205087)
Supplement: Supplementary file 1 — Supporting Information [file ADVS-10-2205087-s001.pdf]

ADVANCED SCIENCE
Open Access

Supporting Information

for *Adv. Sci.*, DOI 10.1002/advs.202205087

Architecture Design and Catalytic Activity: Non-Noble Bimetallic CoFe/fe$_3$O$_4$ Core–Shell Structures for CO_2 Hydrogenation

Wenkang Miao, Ronghui Hao, Jingzhou Wang, Zihan Wang, Wenxin Lin, Heguang Liu, Zhenjie Feng, Yingchun Lyu, Qianqian Li, Dongling Jia*, Runhai Ouyang*, Jipeng Cheng, Anmin Nie and Jinsong Wu*

Supporting Information

Architecture Design and Catalytic Activity: Non-noble Bimetallic CoFe/Fe_3O_4 Core-Shell Structures for CO_2 Hydrogenation

Wenkang Miao, Ronghui Hao, Jingzhou Wang, Zihan Wang, Wenxin Lin, Heguang Liu, Zhenjie Feng, Yingchun Lyu, Qianqian Li, Dongling Jia*, Runhai Ouyang*, Jipeng Cheng, Anmin Nie, Jinsong Wu*

Wenkang Miao, Ronghui Hao, Jingzhou Wang, Zihan Wang, Zhenjie Feng, Yingchun Lyu, Qianqian Li* Runhai Ouyang*

Materials Genome Institute, Shanghai University, Shanghai 200444, China
E-mail: qianqianli@shu.edu.cn; rouyang@shu.edu.cn

Wenxin Lin

School of Materials Science and Engineering, Zhejiang Sci-Tech University, Hangzhou, 310018, China.

Heguang Liu

School of Materials Science and Engineering, Xi'an University of Technology, Xi'an 710048, China

Dongling Jia

Collaborative Research Center, Shanghai University of Medicine & Health Sciences, Shanghai 201318, China

E-mail: jiadongling1@126.com

Jipeng Cheng

School of Materials Science and Engineering, Zhejiang University, Hangzhou 310027, China

Anmin Nie

Center for High Pressure Science, State Key Laboratory of Metastable Materials Science and Technology, Yanshan University, Qinhuangdao 066004, China

Jinsong Wu
Nanostructure Research Center, Wuhan University of Technology, Wuhan, 430070, China

Experimental Section

Synthesis of g-C_3N_4 Two-dimensional nanosheet. First, specifically: put the precursor urea (10 g) containing carbon and nitrogen into a 50 ml crucible, cover it, put it into a muffle furnace, and heat up to 550°C at a heating rate of 2.3°C/min in the air. After 4 h, the temperature was lowered to room temperature at a rate of 1°C/min to obtain bulk g-C_3N_4, the obtained bulk g-C_3N_4 was placed in a crucible without a lid, and heated to 500°C in air at a rate of 5°C/min, stored for 2 h, and then lowered to room temperature to obtain porous g-C_3N_4 powder. Then, a certain amount of porous g-C_3N_4 powder was dispersed in deionized water, ultrasonically pulverized for 30 min with a probe-type ultrasonic pulverizer with a power of 300 W, and centrifuged at 3000 rpm for 10 min to remove the precipitate, and the upper dispersion liquid was collected and freeze-dried, to obtain two-dimensional g-C_3N_4 nanosheets.

Synthesis of $Co_xFe_yO_4$[1]. 0.5 mmol (128 mg) cobalt acetylacetonate and 1 mmol (353 mg) iron acetylacetonate were ultrasonically dispersed in 6 g triethylene glycol. The mixture was first heated to 110°C for 30 min to remove low-boiling impurities, resulting in a clear red solution. The temperature was then increased to 250°C and held at this temperature for 60 min. The solution was cooled to room temperature, 10 mL of ethyl acetate was added to the cooled solution to precipitate nanoparticles, followed by centrifugation and repeated washes with ethyl acetate for several times. The final product was redispersed in water and freeze-dried to obtain $CoFe_2O_4$ nanoparticles. The synthesis of $Co_xFe_{2-x}O_4$ adopts the same preparation process as $CoFe_2O_4$. The total molar ratio of metal precursors remains unchanged. The molar ratios of cobalt acetylacetonate and iron acetylacetonate are changed to 2:1, 1.5:1, and 1:1, respectively.

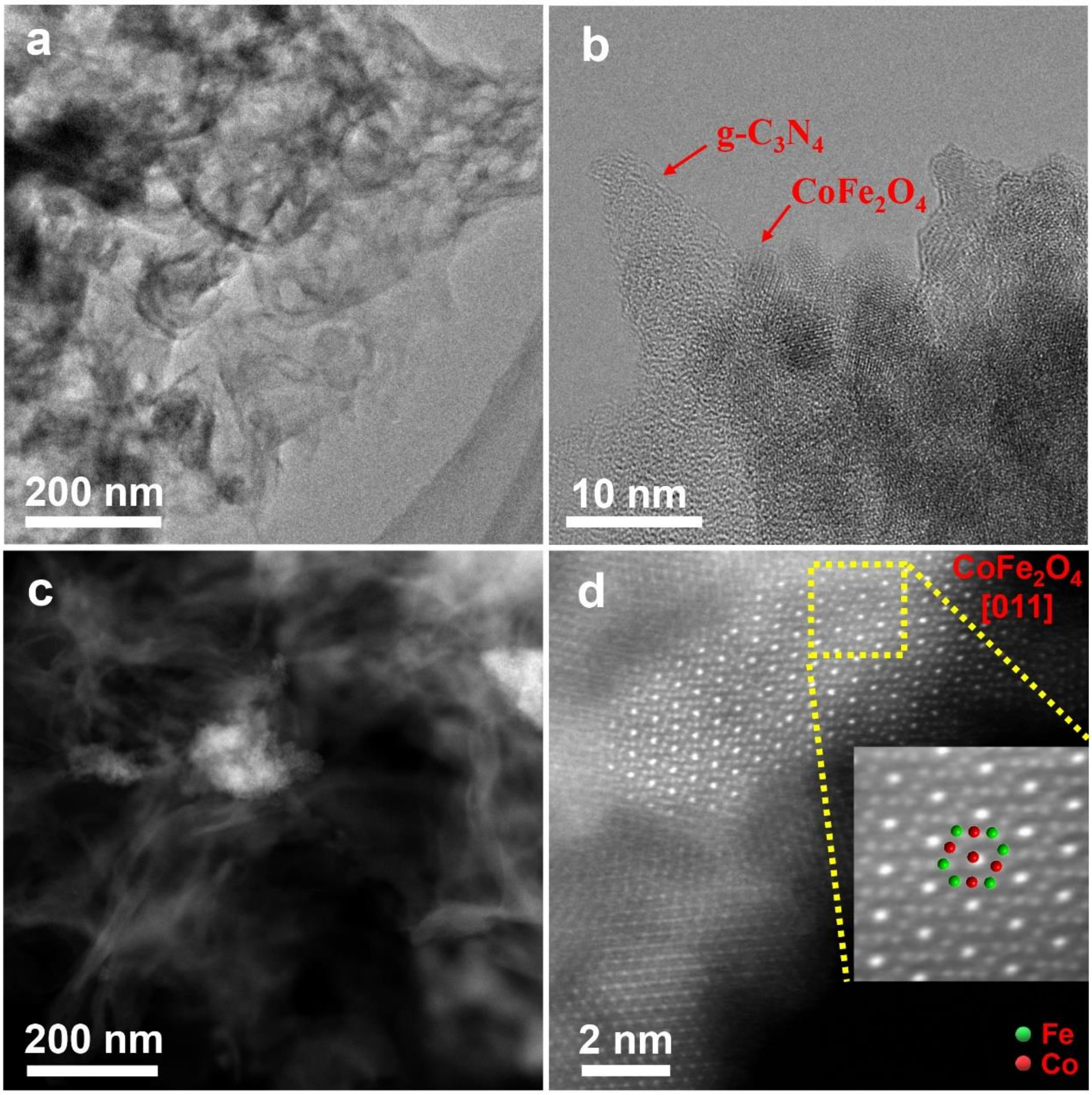

Figure S1. Characterization of g-C_3N_4/$CoFe_2O_4$-15wt%. a, b) TEM images and c, d) AC-STEM images.

Notes:

Spinel cobalt iron oxide nanoparticles with different atomic ratios of Co and Fe were uniformly anchored on. As the Co/Fe ratio was fixed as 1:2 in precursors, the final products could be indexed as spinel $CoFe_2O_4$, indicated by the atomic resolution TEM images in Figure S1d. The average size of particles was measured to 5 nm.

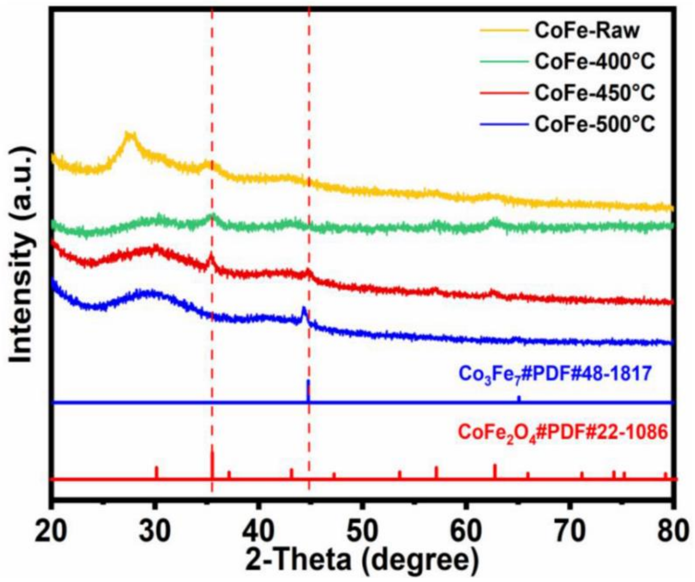

Figure S2. Phase of different temperatures. The XRD patterns of CoFe with different pretreatment temperatures.

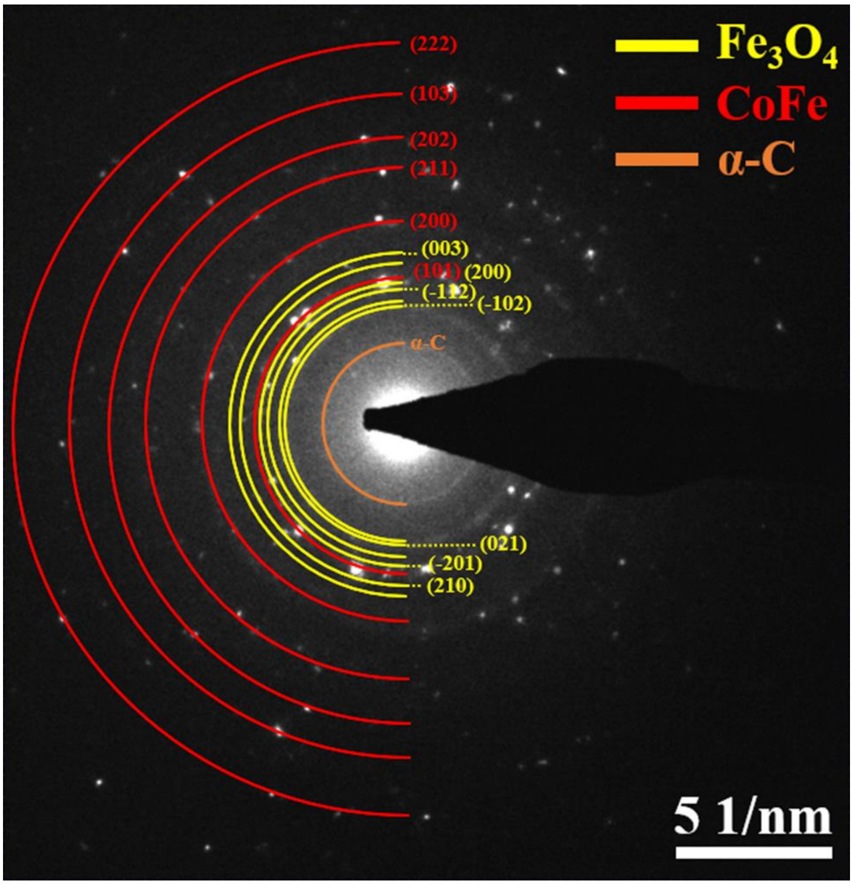

Figure S3. Characterization of CoFe (1:1) (CoFe/Fe_3O_4). An enlarged view of the selected electron diffraction is shown in the illustration of Fig. 2a.

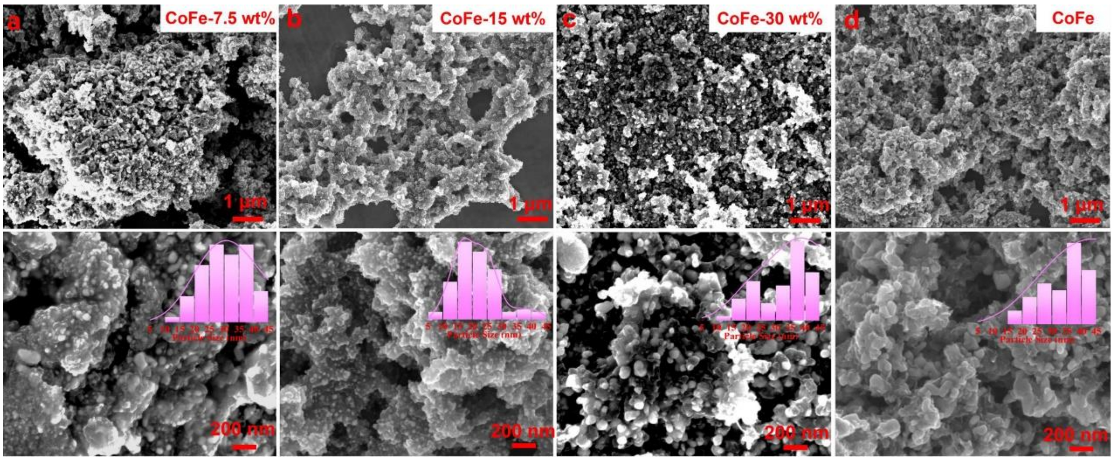

Figure S4. Characterization of morphology. The SEM image of CoFe with different CoFe loading.

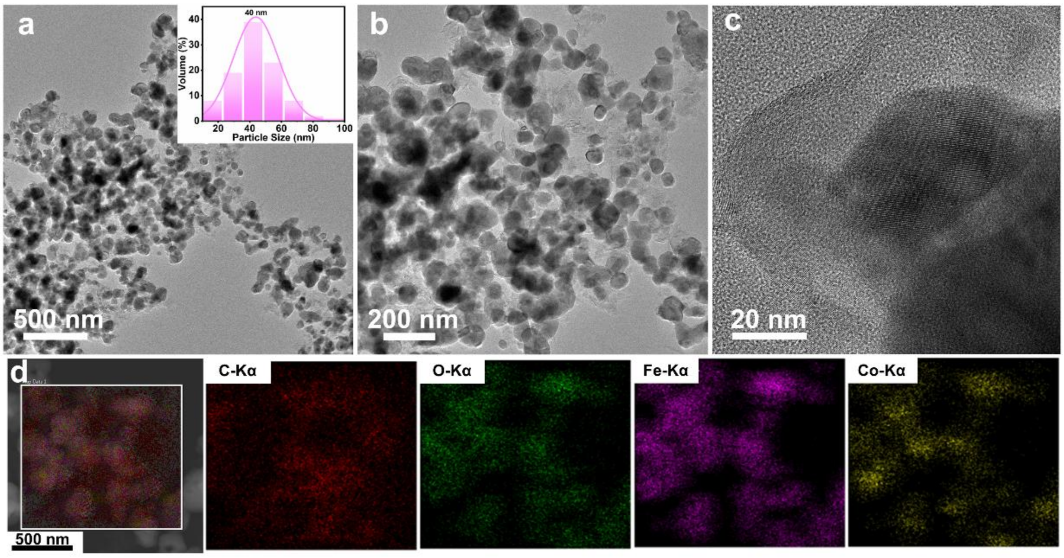

Figure S5. Characterization of TEM with different loading amount. a-c) Low to high magnification TEM images of the α-CoFe-7.5 wt%, the insets show particle size distribution in (a). d) EDS mapping.

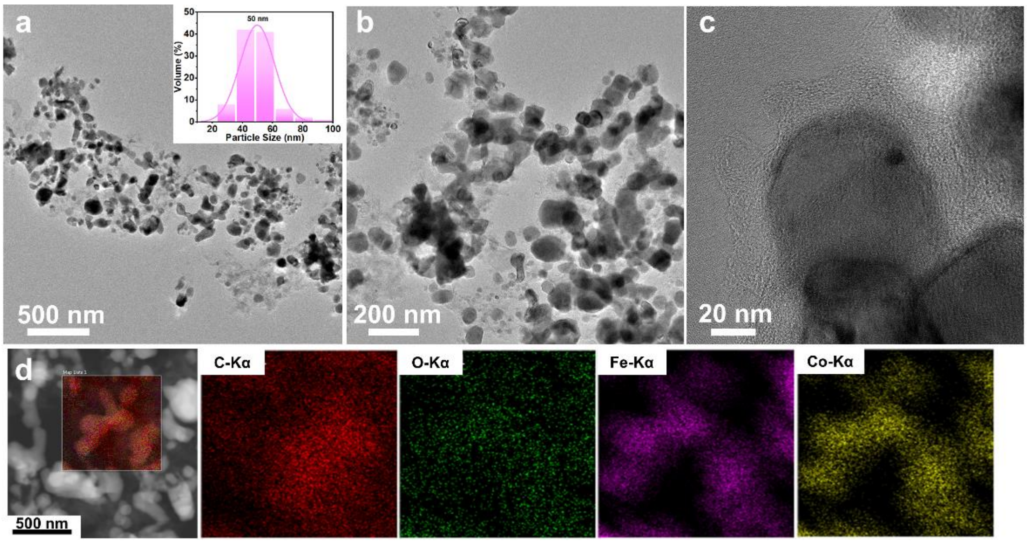

Figure S6. Characterization of TEM with different loading amount. a-c) Low to high magnification TEM images of the α-CoFe-30 wt%, the insets show particle size distribution in (a). d) EDS mapping.

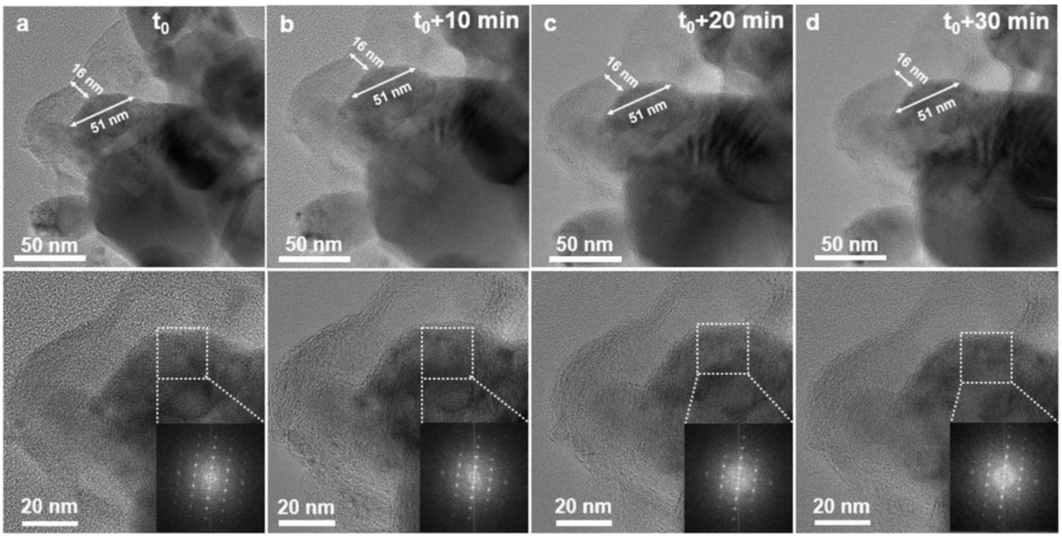

Figure S7. Electron beam radiation damage test. a-d) TEM images of different electron beam irradiation times, from 0-30 min, the following figures are the corresponding high-resolution images, and the inset is the FFT in the dotted area.

Notes:

We tested the damage effect of electron beam irradiation on the sample, the results are shown in Figure S7. The electron beam irradiation area includes carbon support and nanoparticles. With the increase of irradiation time, the thickness of exposed carbon support and the diameter of nanoparticles have not changed, which

are 16 nm and 51 nm, respectively. The phase of nanoparticles in the corresponding FFT has not changed, so we conclude that the electron beam irradiation will not damage the sample in morphology and phase.

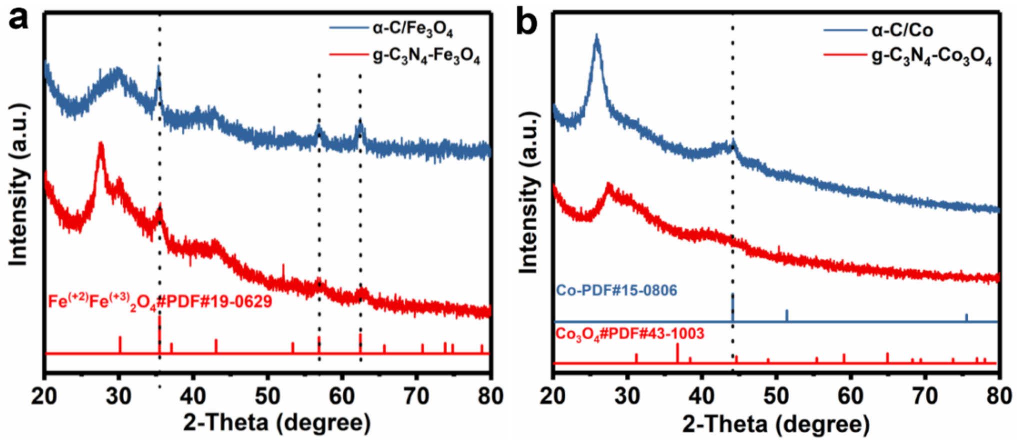

Figure S8. Phase of various catalyst. The XRD patterns of a) Fe_3O_4 and b) Co_3O_4.

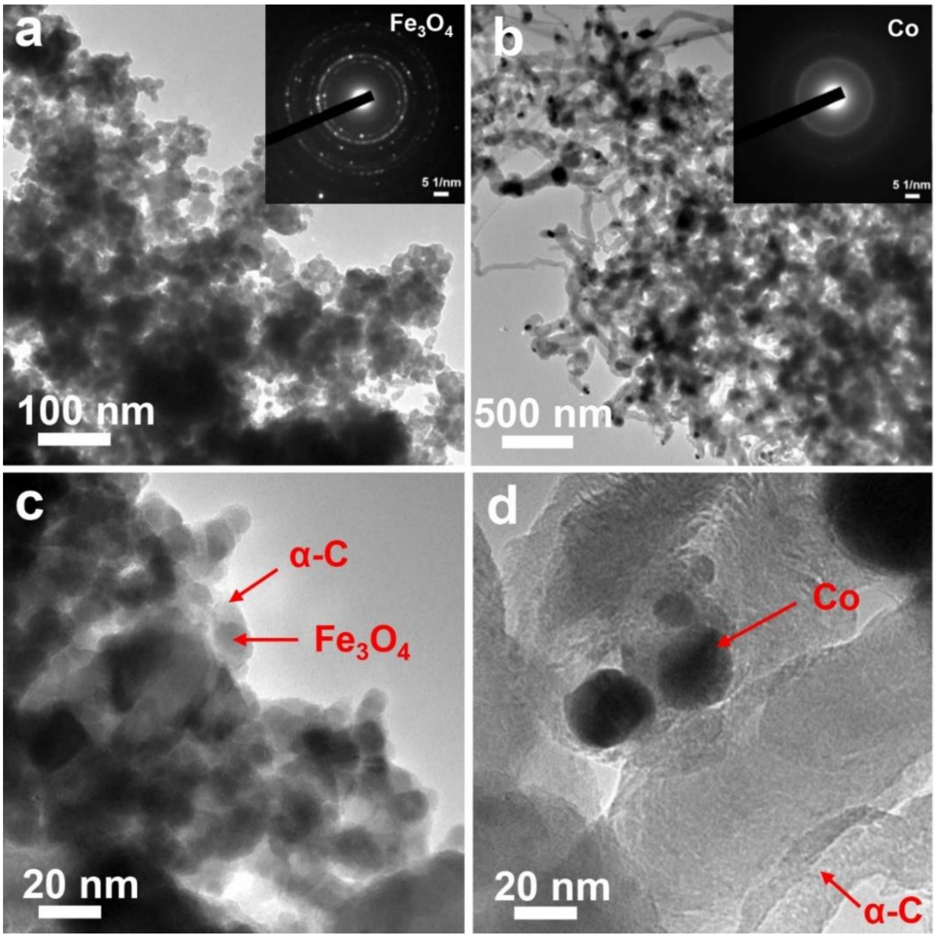

Figure S9. Characterization of morphology. The TEM image of a, c) α-C/Co and b, d) α-C/Fe_3O_4.

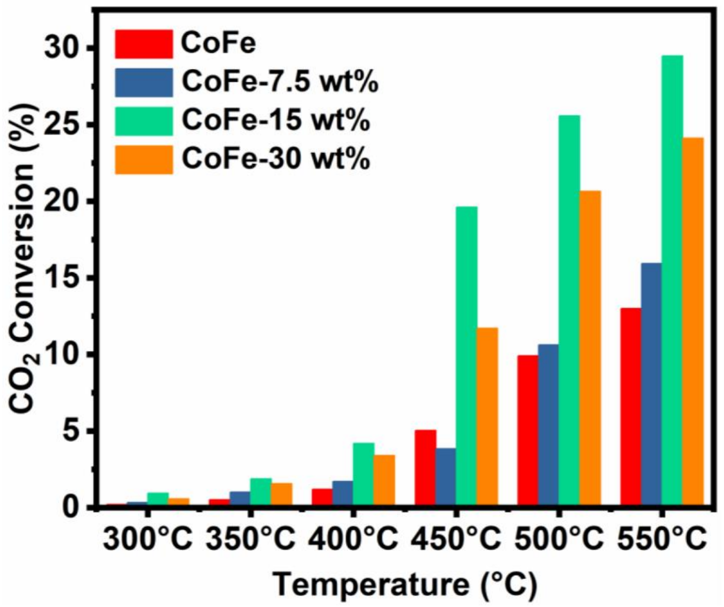

Figure S10. Characterization of catalytic activity. The performance of different loads at different work temperatures

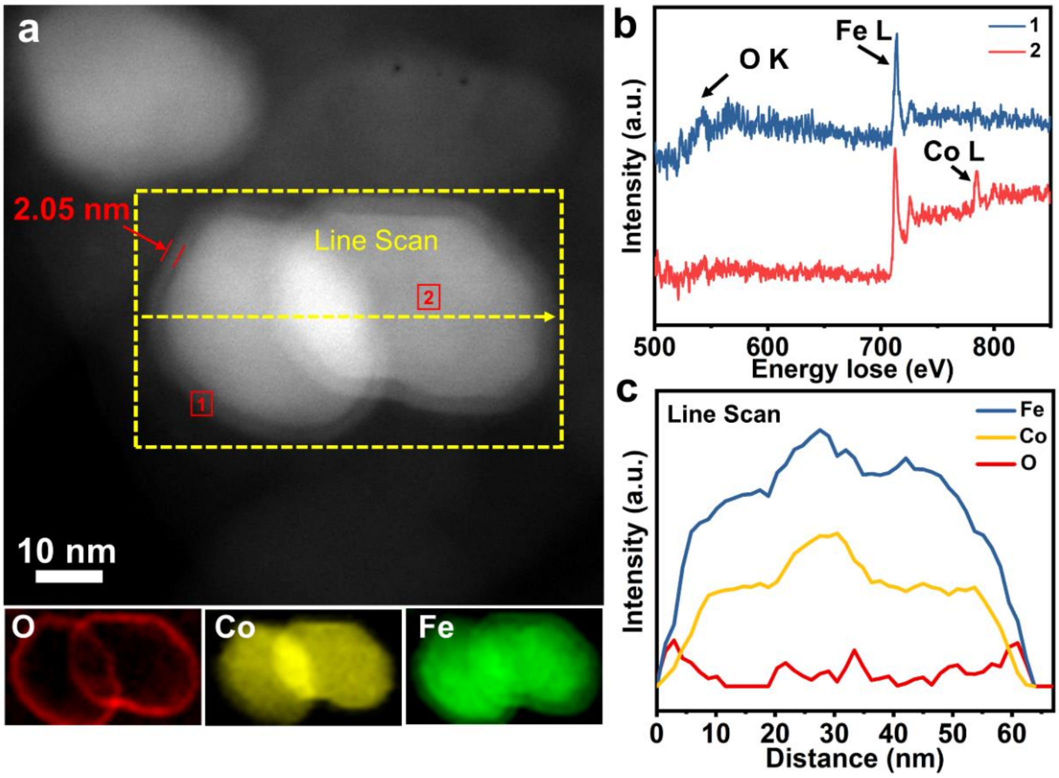

Figure S11. The morphology and core-shell composition of the catalyst after 90 h stability test. a) EELS mapping of CoFe (1:1) after 90 h reaction and b) Core-loss spectrums of O *K*-edge, Fe *L*-edge and Co *L*-dege. c) EELS line scan spectrum (the yellow dotted lines showing the line scan trace).

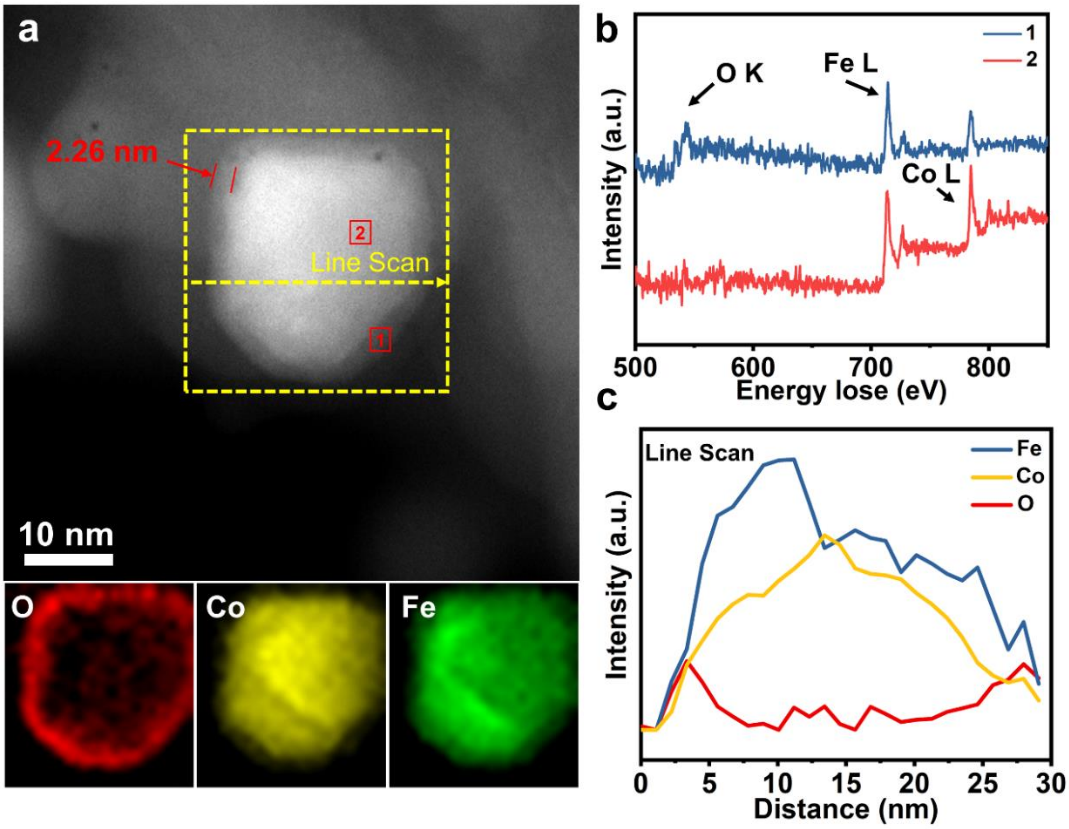

Figure S12. The morphology and core-shell composition of the catalyst after 90 h stability test and fully oxidized in air. a) EELS mapping of CoFe (1:1) after 90 h reaction and fully oxidized in air. b) Core-loss spectrums of O *K*-edge, Fe *L*-edge and Co *L*-dege. c) EELS line scan spectrum (the yellow dotted lines showing the line scan trace).

Notes:

In order to confirm once again the reversible exsolution/dissolution of Co in the shell and the stability of the catalyst, we characterized the morphology and elemental composition of the catalyst after 90h reaction, as shown in Figure S11 and Figure S12. Co was not detected in the shell just after the reaction (Figure S11), but when the catalyst was fully exposed to the air, partially Co migrated from the core to the shell to form a spinel structure, accompanied by the thickening of the oxide layer (Figure S12.).

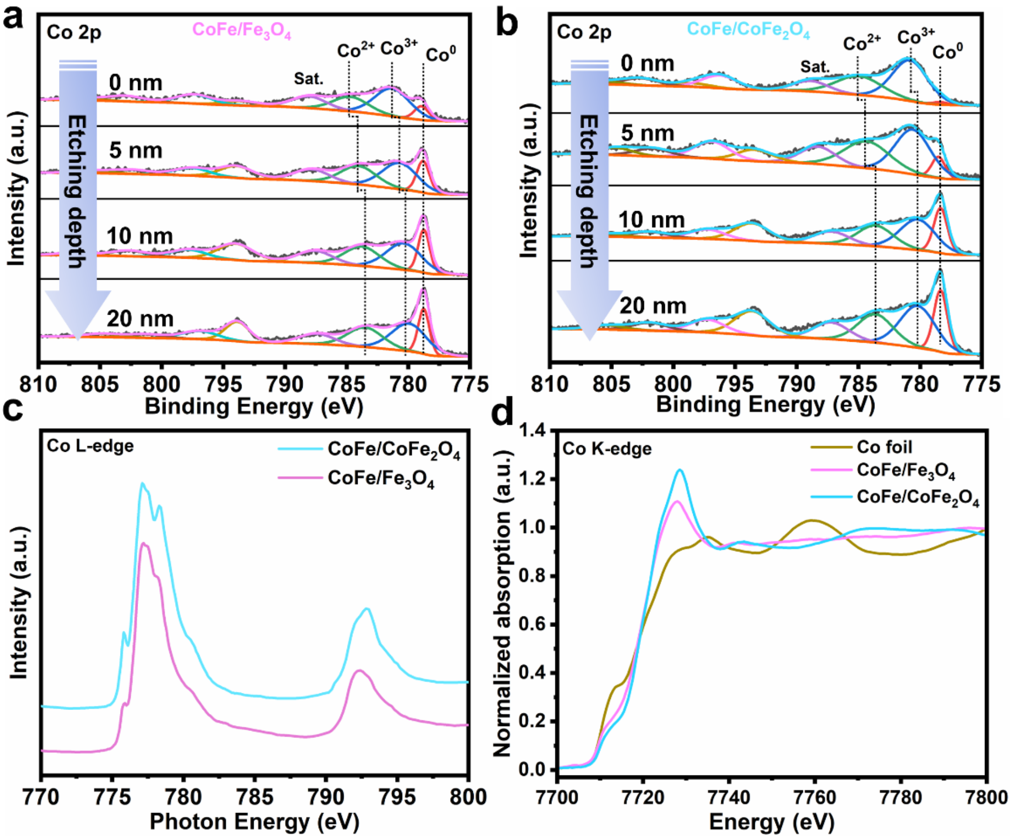

Figure S13. Electronic structural characterization of activity degradation and regeneration. a, b) The high-resolution Fe 2p (a) and Co 2p (b) XPS depth profiling of $CoFe/CoFe_2O_4$. c) XAFS of Co L-edge. d) XANES of normalized Co K-edge.

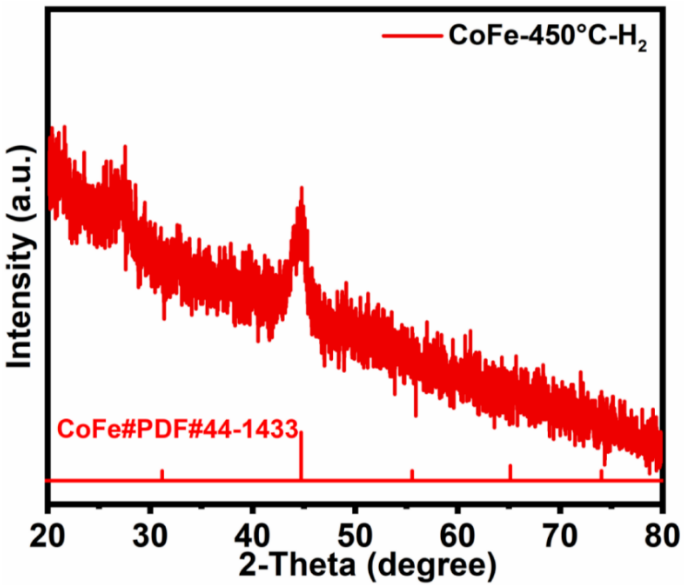

Figure S14. Regulation of core-shell structural components. The XRD patterns of CoFe (1:1) - H_2

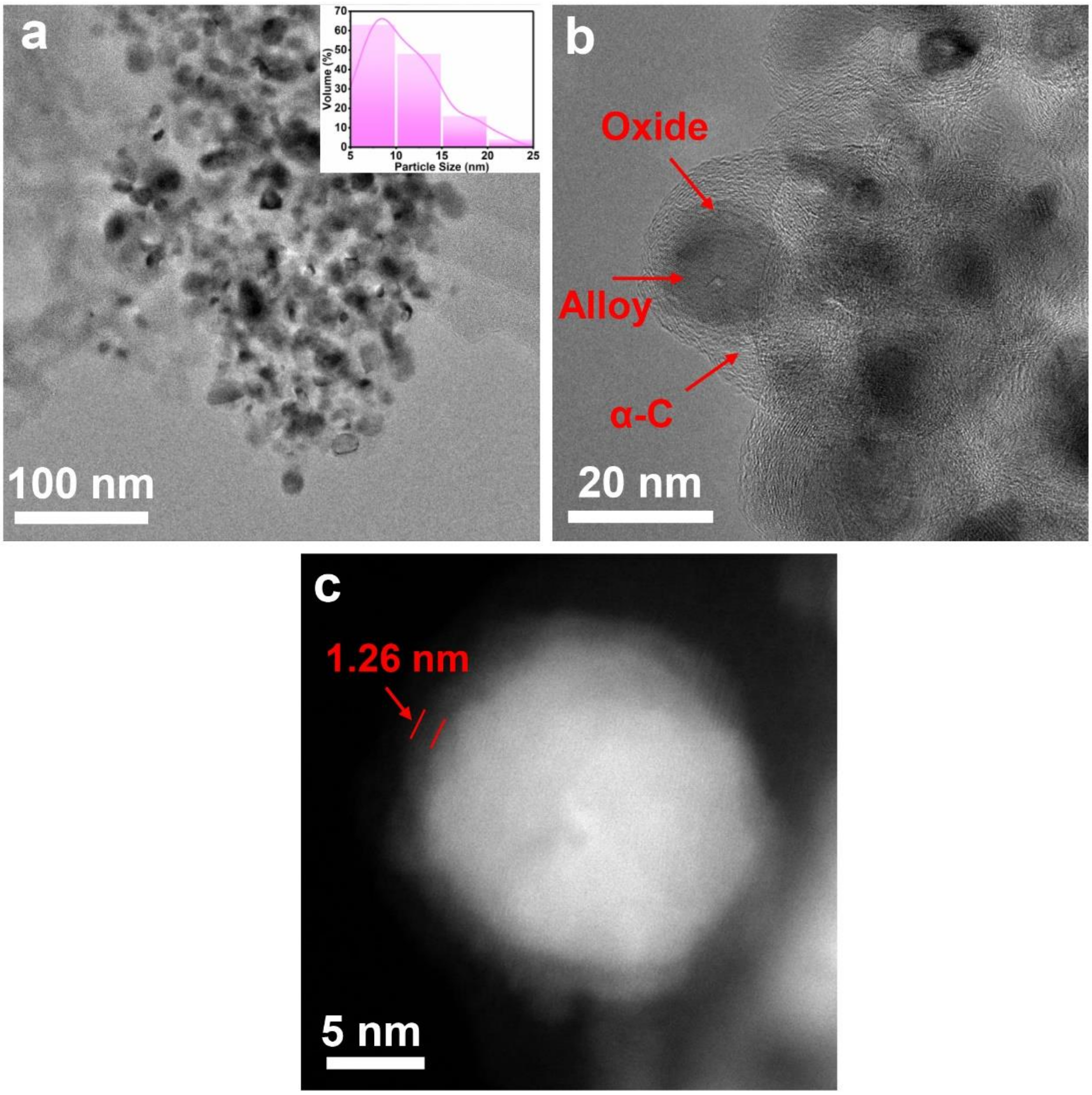

Figure S15. Regulation of core-shell structural components. a, b) TEM images of CoFe (1:1) - H_2 and c) AC-STEM images.

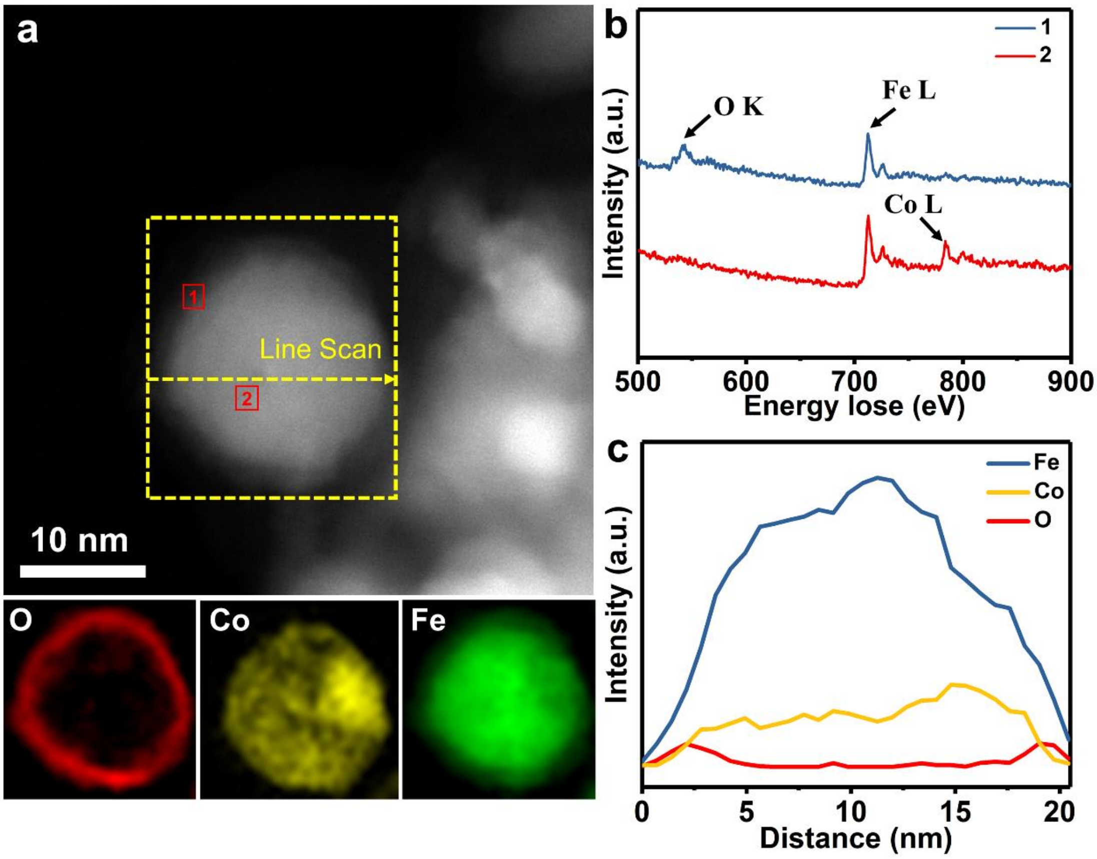

Figure S16. Regulation of core-shell structural components. a) EELS mapping of CoFe (1:1) - H_2 and (b) Core-loss spectrums of O K-edge, Fe *L*-edge and Co *L*-edge. c) EELS line scan spectrum (the yellow dotted lines showing the line scan trace).

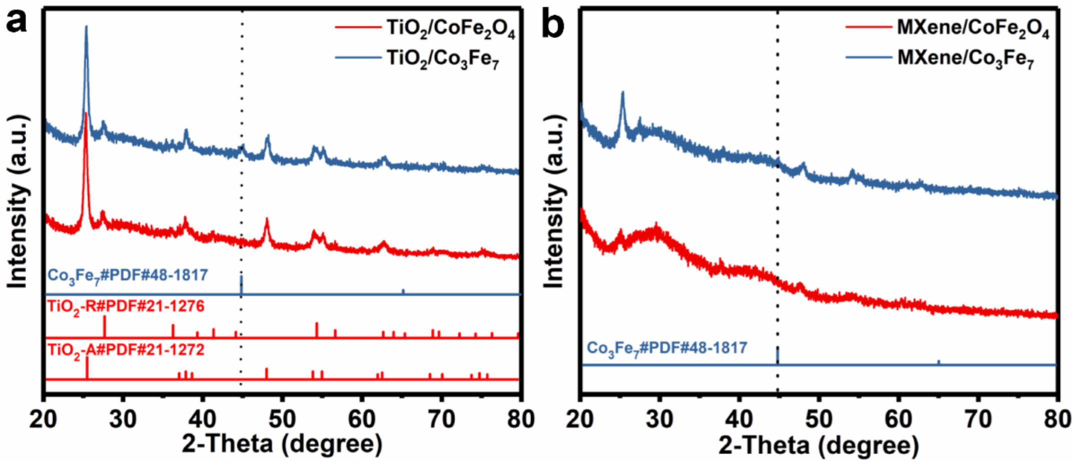

Figure S17. Phase of various supports. a) TiO_2 and b) MXene.

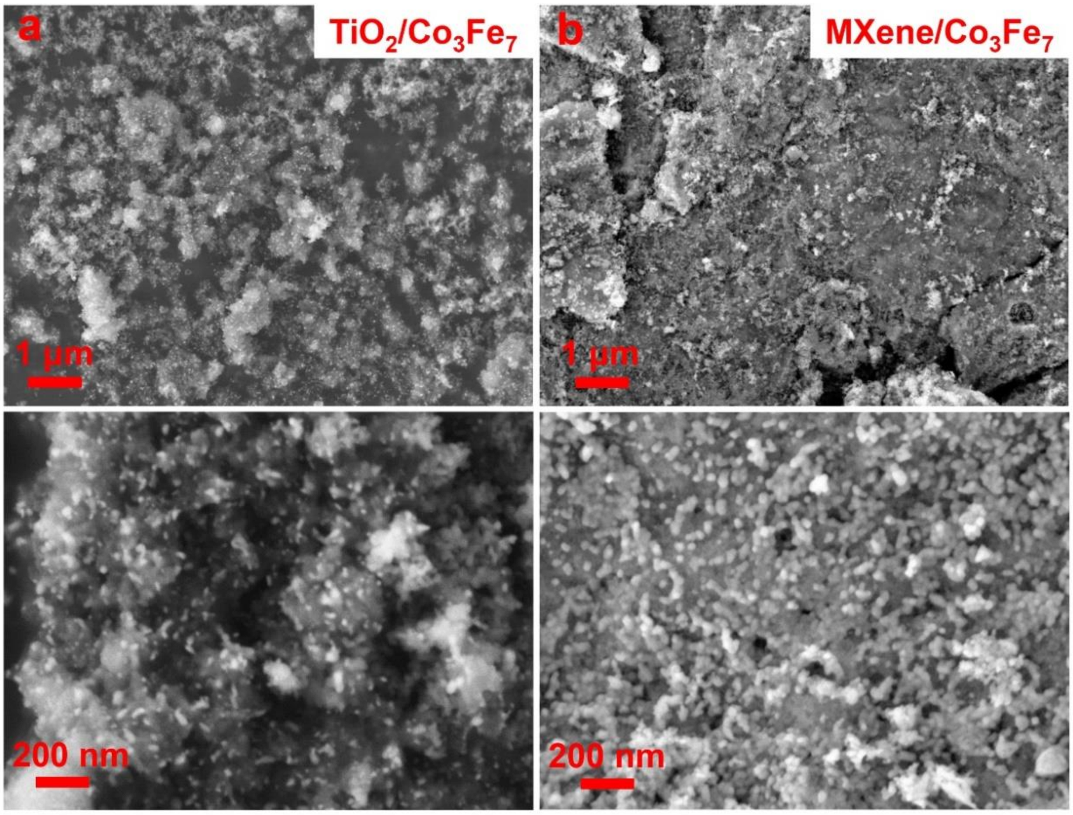

Figure S18. Morphology of various supports. The SEM image of TiO_2/Co_3Fe_7 and $MXene/Co_3Fe_7$.

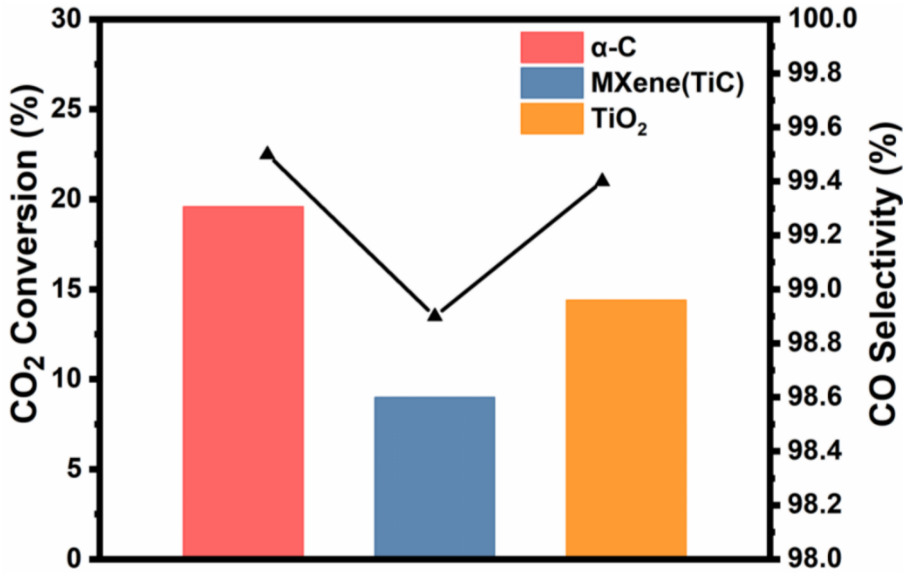

Figure S19. Catalytic activity of various supports. CO_2 hydrogenation activity of various supports, the stick represents CO_2 conversion and the dot-line graph represents CO selectivity.

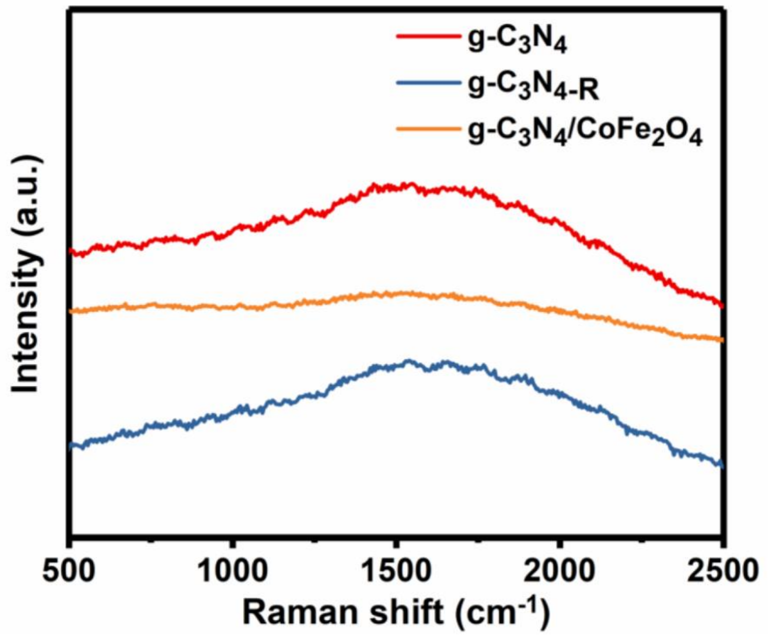

Figure S20. Regulation of the supports. The Raman spectra of g-C_3N_4.

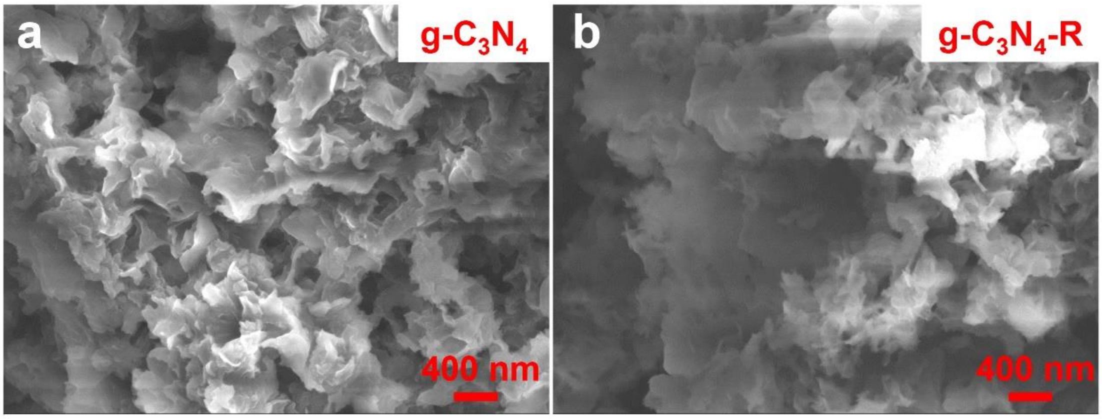

Figure S21. Characterization of morphology. The SEM image of g-C_3N_4.

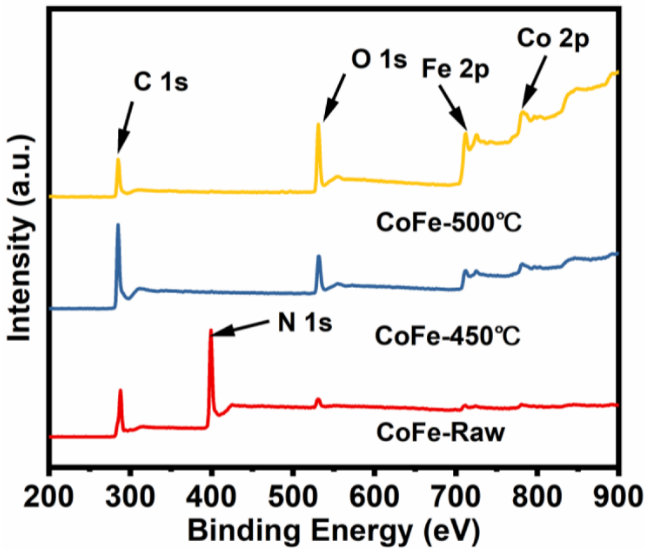

Figure S22. Characterization of electronic structure. XPS spectra of α-C pretreated at 450°C and 500°C

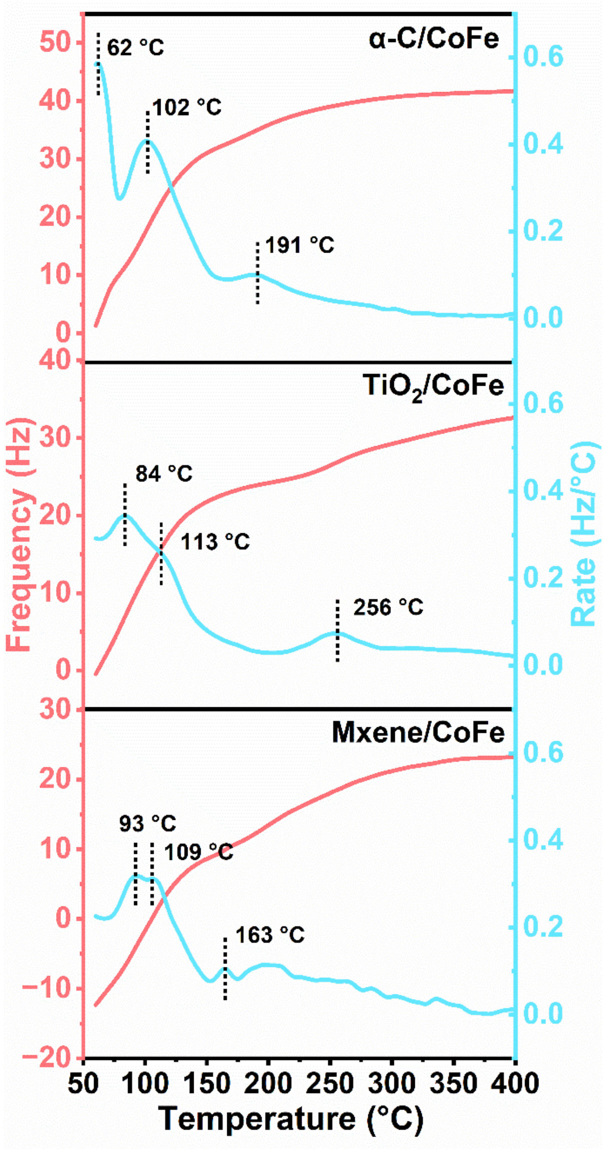

Figure S23. Characterization of CO_2-TPD with different kinds of supports.

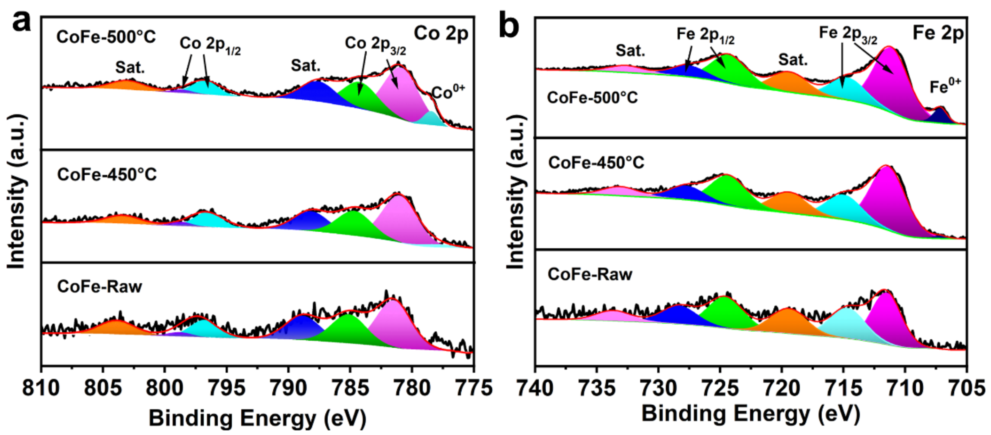

Figure S24. Characterization of electronic structure. High-resolution XPS spectra of a) Co 2p and b) Fe 2p.

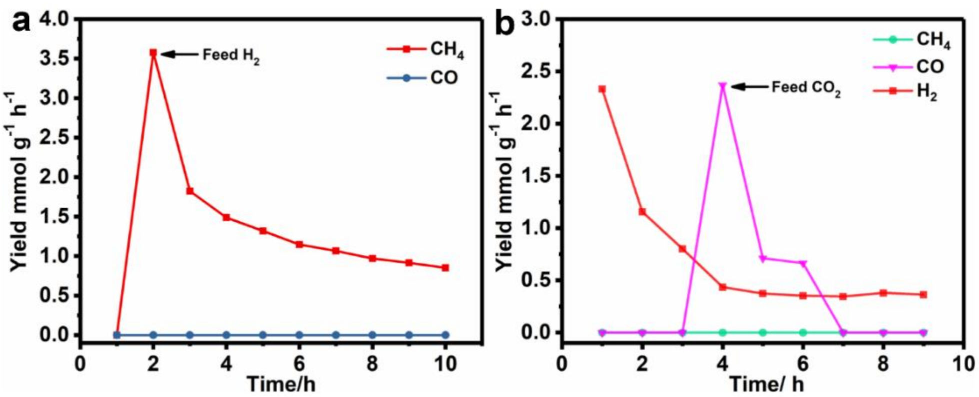

Figure S25. Catalytic reaction mechanism. The products under different pretreatment conditions of CoFe (1:1).

We hope to gain insight into the reaction process through different reaction conditions. Figure S20a. shows that the sample was treated in a pure CO_2 environment, then the CO_2 in the reactor was removed with Ar, and H_2 began to be fed until CO_2 had been completely removed. We detected a large amount of CH_4 but no CO. According to the results of XPS, we speculate that plenty of CO_2 would be adsorbed on the surface of the catalyst and hydrogenated to CH_4 when hydrogen is fed into the reaction process. However, the selectivity tends to CH_4 due to the lack of hydrogen assisted in the CO_2 activation process, resulting in the production of intermediates conducive to methanation. Similarly, Figure S20b. implies that the sample was treated in a pure H_2 environment, then H_2 in the reactor was removed with Ar, and CO_2 began to be fed when H_2 was completely removed. Differently, we detected a large amount of CO, at the same time, the yield of CH_4 was 0. Combined with the above experimental conclusions, we sum up that different products may be caused by different kinetic mechanisms: (1) selective CH_4 production tends to the L-H mechanism, that is, CO_2 and H_2 molecules are adsorbed on the carbon support and metal respectively, then producing CH_4 on the surface after totally completed reaction; (2) Selective CO production tends to E-R reaction mechanism, that is, H_2 is dissociated and spilled out on the metal surface, which then assists in the activation and conversion of CO_2 on the defective carbon support. Under the reaction conditions of CO_2 hydrogenation, E-R reaction mechanism is dominant, thus exhibiting the excellent CO selectivity.

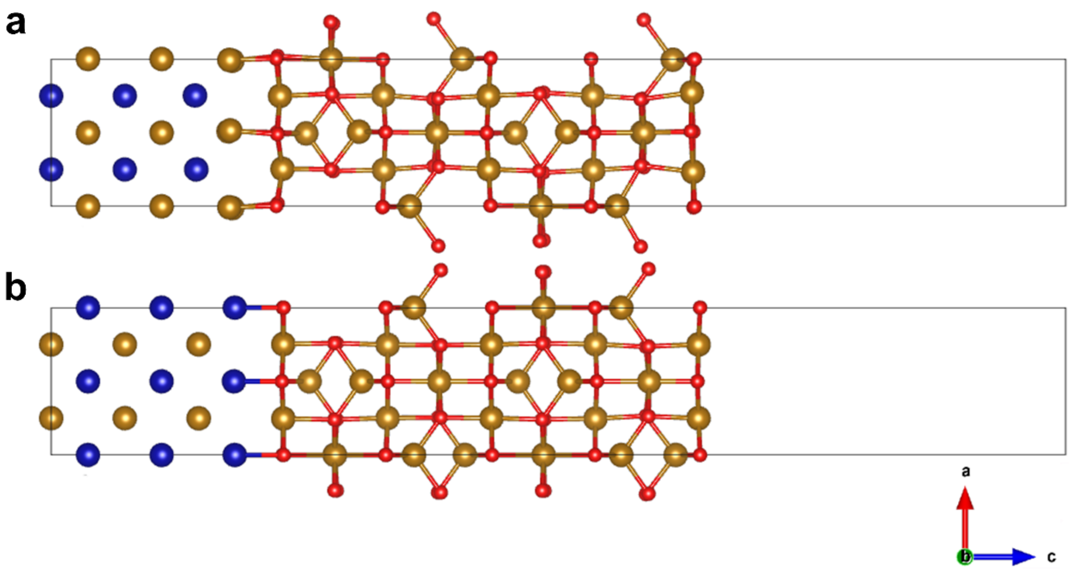

Figure S26. Slab model. a) $CoFe/Fe_3O_4$ and b) $FeCo/Fe_3O_4$.

Supplementary tables

Table S1. Effect of reversible dissolution of Co on crystal plane interplanar spacing of alloy core. The dissolution of Co leads to expansion.

CoFe	Theoretical value	Observed value	expansion rate
(110)	2.02 Å	2.14 Å	106.0%
(200)	1.43 Å	1.52 Å	106.3%
(1-10)	2.02 Å	2.13 Å	105.4%

Table S2. Comparison between α-C-CoFe/Fe_3O_4 and other reported catalyst activities.

Samples	H_2:CO_2 ratio	Temperature (°C)	WHSV ($ml\cdot g_{cat}^{-1}h^{-1}$)	CO_2 Conversion (%)	CO selectivity (%)	Ref
CoFe/Fe_3O_4	1:1	450	30000	29	99	This work
Pt-Co/CeO_2	2:1	300	60000	9	92	[2]
PD-In/SiO_2	1:1	600	60000	10	100	[3]
Cu/β-Mo_2C	4:1	450	12000	30	100	[4]
Co-CeO_2	1:1	600	600000	35	98.5	[5]
Ru-CeO_2	1:1	600	120000	38	100	[6]
NiCu-Sap	4:1	600	15000	53	89	[7]
NiFe/CeAl	4:1	650	30000	68	80	[8]

Table S3. Effect of reversible dissolution of Co on crystal plane interplanar spacing of alloy core. The dissolution of Co slows the expansion.

CoFe	Theoretical value	Observed value	expansion rate
(110)	2.02 Å	2.04 Å	101.0%
(200)	1.43 Å	1.45 Å	101.4%
(1-10)	2.02 Å	2.05 Å	101.5%

Table S4. The proportion of Fe and Co in different valence states.

$CoFe/Fe_3O_4$	Fe^0	Fe^{2+}	Fe^{3+}	Co^0	Co^{2+}	Co^{3+}
0 nm	7.08	61.41	31.51	11.51	30.50	57.99
5 nm	11.28	51.54	37.19	20.25	33.08	46.67
10 nm	13.97	48.31	37.72	23.24	32.40	44.36
20 nm	18.20	46.14	35.66	23.05	31.90	45.05
**$CoFe/CoFe_2O_4$**	**Fe^0**	**Fe^{2+}**	**Fe^{3+}**	**Co^0**	**Co^{2+}**	**Co^{3+}**
0 nm	2.41	59.71	39.68	1.69	36.76	61.56
5 nm	6.23	52.94	40.83	9.17	39.73	51.10
10 nm	11.84	48.00	40.16	18.11	30.27	51.62
20 nm	15.11	38.45	46.44	25.13	30.34	44.53

Table S5. Effect of reversible dissolution of Co on crystal plane interplanar spacing of alloy core.

CoFe	Theoretical value	Observed value	expansion rate
(1-1-2)	1.17 Å	1.14 Å	97.4%
(1-10)	2.02 Å	1.98 Å	98.0%
(-11-2)	1.17 Å	1.16 Å	99.0%

Table S6. Adsorption energy ($\Delta E_{\mathrm{ads}}^{\mathrm{H}}$, eV) of H atom and formation energy of O vacancy ($\Delta E_{\mathrm{vac}}^{\mathrm{O}}$, eV) on different site of the oxide surfaces. The "FeCo" and "CoFe" are the same alloy but with Co and Fe termination at the interface, respectively (Figure S26, Supporting Information). Fe_3O_4 (expanded) denotes the expanded oxide detached from the $CoFe/Fe_3O_4$ structure, and Fe_3O_4 (normal) denotes the oxide that has the lattice constant as that in perfect crystal.

Systems	$FeCo/Fe_3O_4$	$CoFe/Fe_3O_4$	Fe_3O_4(expanded)	Fe_3O_4(normal)	$CoFe_2O_4$
$\Delta E_{\mathrm{ads-3}}^{\mathrm{H}}$	-2.78	-2.82	-2.77	-2.83	-3.54
$\Delta E_{\mathrm{ads-4}}^{\mathrm{H}}$	-3.37	-3.47	-3.44	-3.40	-3.95
$\Delta E_{\mathrm{ads-1}}^{\mathrm{H}}$	-	-	-1.78	-	-
$\Delta E_{\mathrm{ads-2}}^{\mathrm{H}}$	-	-	unstable	-	-
$\Delta E_{\mathrm{vac-3}}^{\mathrm{O}}$	5.79	5.81	5.79	5.94	5.43
$\Delta E_{\mathrm{vac-4}}^{\mathrm{O}}$	5.04	5.04	5.08	5.15	4.67

Supplementary References

[S1] A. El Arrassi, Z. Liu, M. V. Evers, N. Blanc, G. Bendt, S. Saddeler, D. Tetzlaff, D. Pohl, C. Damm, S. Schulz, K. Tschulik, *J. Am. Chem. Soc.* **2019**, *141*, 9197.

[S2] S. Kattel, W. Yu, X. Yang, B. Yan, Y. Huang, W. Wan, P. Liu, J. G. Chen, *Angew. Chem. Int. Ed.* **2016**, *55*, 7968.

[S3] J. Ye, Q. Ge, C. Liu, *Chem. Eng. Sci.* **2015**, *135*, 193.

[S4] Q. Zhang, L. Pastor-Pérez, W. Jin, S. Gu, T. R. Reina, *Appl. Catal. B Environ.* **2019**, *244*, 889.

[S5] Y. He, K. R. Yang, Z. Yu, Z. S. Fishman, L. A. Achola, Z. M. Tobin, J. A. Heinlein, S. Hu, S. L. Suib, V. S. Batista, L. D. Pfefferle, *Nanoscale* **2019**, *11*, 16677.

[S6] C. Panaritis, M. Edake, M. Couillard, R. Einakchi, E. A. Baranova, *J. CO2 Util.* **2018**, *26*, 350.

[S7] N. Nityashree, C. A. H. Price, L. Pastor-Perez, G. V. Manohara, S. Garcia, M. M. Maroto-Valer, T. R. Reina, *Appl. Catal. B Environ.* **2020**, *261*, 118241.

[S8] L. Yang, L. Pastor-Pérez, S. Gu, A. Sepúlveda-Escribano, T. R. Reina, *Appl. Catal. B Environ.* **2018**, *232*, 464.
